# Resonance as a Design Strategy for AI and Social Robots

**DOI:** 10.3389/fnbot.2022.850489

**Published:** 2022-04-27

**Authors:** James Derek Lomas, Albert Lin, Suzanne Dikker, Deborah Forster, Maria Luce Lupetti, Gijs Huisman, Julika Habekost, Caiseal Beardow, Pankaj Pandey, Nashra Ahmad, Krishna Miyapuram, Tim Mullen, Patrick Cooper, Willem van der Maden, Emily S. Cross

**Affiliations:** ^1^Department of Human Centered Design, Faculty of Industrial Design Engineering, Delft University of Technology, Delft, Netherlands; ^2^Center for Human Frontiers, Qualcomm Institute, University of California, San Diego, San Diego, CA, United States; ^3^Department of Psychology, New York University, New York, NY, United States; ^4^Department of Clinical Psychology, Vrije Universiteit Amsterdam, Amsterdam, Netherlands; ^5^The Design Lab, California Institute of Information and Communication Technologies, University of California, San Diego, San Diego, CA, United States; ^6^Centre for Cognitive and Brain Sciences, Indian Institute of Technology, Gandhinagar, India; ^7^Intheon Labs, San Diego, CA, United States; ^8^Department of Physics, Duquesne University, Pittsburgh, PA, United States; ^9^Social Robotics, Institute of Neuroscience and Psychology, School of Computing Science, University of Glasgow, Glasgow, United Kingdom; ^10^SOBA Lab, School of Psychology, Macquarie University, Sydney, NSW, Australia

**Keywords:** resonance, entrainment, synchronization, metaphor, design space, social robotics, AI for wellbeing, human-media interaction

## Abstract

Resonance, a powerful and pervasive phenomenon, appears to play a major role in human interactions. This article investigates the relationship between the physical mechanism of resonance and the human experience of resonance, and considers possibilities for enhancing the experience of resonance within human–robot interactions. We first introduce resonance as a widespread cultural and scientific metaphor. Then, we review the nature of “sympathetic resonance” as a physical mechanism. Following this introduction, the remainder of the article is organized in two parts. In part one, we review the role of resonance (including synchronization and rhythmic entrainment) in human cognition and social interactions. Then, in part two, we review resonance-related phenomena in robotics and artificial intelligence (AI). These two reviews serve as ground for the introduction of a design strategy and combinatorial design space for shaping resonant interactions with robots and AI. We conclude by posing hypotheses and research questions for future empirical studies and discuss a range of ethical and aesthetic issues associated with resonance in human–robot interactions.

## Introduction

Resonance is a powerful physical mechanism that manifests in any physical system involving oscillations (Buchanan, 2019). Examples include the electromagnetic resonances that enable wireless communications, the acoustic resonances that give musical instruments their beauty, and the orbital resonances that shaped our solar system. No matter the medium, resonance produces amplification and synchronization effects in oscillatory systems. Details on the varying kinds of resonance are found in Box 1.

Box 1 **A compilation of “resonance” terms from the scientific literature.**

The following table outlines the breadth of the concept of resonance across three domains in the social and physical sciences. The examples given for each type of resonance (right column) are not meant to be an exhaustive reference list—instead, our intention is to include a few illustrative examples of each conception of resonance.

**Table:** 

**Psychology and neuroscience**	
**Affective resonance**	**Decety, 2010; Mühlhoff, 2019**
**Bodily resonance**	**Bedder et al., 2019**
**Conceptual resonance**	**Lee et al., 2007; Howie and Bagnall, 2020**
**Cognitive resonance**	**Giorgi, 2017**
**Embodied resonance**	**Kirsch et al., 2016b; Gallese and Sinigaglia, 2018**
**Emotional resonance**	**Gratch et al., 2013; Schrock et al., 2004; Decety, 2010; Giorgi, 2017**
**Empathic resonance**	**Azevedo et al., 2013**
**Harmonic resonance**	**Lehar, 2003**
**Interpersonal resonance**	**Uithol et al., 2011; Himberg et al., 2018**
**Intrapersonal resonance**	**Uithol et al., 2011**
**Limbic resonance**	**Lewis et al., 2001**
**Motor resonance**	**Cross et al., 2006; Aglioti et al., 2008**
**Neural resonance**	**Large and Snyder, 2009; Katz, 1995**
**Neuroaesthetic resonance**	**Beardow, 2021**
**Perceptual resonance**	**Schütz-Bosbach and Prinz, 2007**
**Physiological resonance**	**Engert et al., 2019**
**Social resonance**	**Kopp, 2010; Wheatley and Sievers, 2016**
**Other social sciences**	
**Advertising resonance**	**McQuarrie and Mick, 1992**
**Aesthetic resonance**	**Farber, 1994**
**Brand resonance**	**Keller, 2010**
**Carnal resonance**	**Paasonen, 2011**
**Consumer resonance**	**Shang et al., 2017**
**Cultural resonance**	**McDonnell et al., 2017**
**Entrepreneurial resonance**	**Warren, 2004**
**Ethical resonance**	**Prasad, 2019**
**Frame resonance**	**Snow and Benford, 1988; Giorgi, 2017**
**Historical resonance**	**Ferreira and Vale, 2020**
**Human resonance**	**Rosa, 2018**
**Interaction resonance**	**Hummels et al., 2003**
**Institutional resonance**	**Strydom, 2003**
**Morphic resonance**	**Sheldrake, 2011**
**Narrative resonance**	**van Werven et al., 2019; Duarte, 2013**
**Norm resonance**	**Gutterman, 2015**
**Political resonance**	**Cunneen, 2019**
**Sexual resonance**	**Baudrillard, 2005**
**Spiritual resonance**	**Siegel, 2013**
**Value resonance**	**Schemer et al., 2012**
**Physics**	
***Types of resonance discussed in physics literature include:***	
**Antiresonance**	**Rajasekar and Sanjuan, 2016**
**Autoresonance**	**Rajasekar and Sanjuan, 2016**
**Chaotic resonance**	**Rajasekar and Sanjuan, 2016**
**Coherence resonance**	**Rajasekar and Sanjuan, 2016**
**Ghost resonance**	**Rajasekar and Sanjuan, 2016**
**Harmonic resonance**	**Li et al., 2020**
**Multiple harmonic resonance**	**Ludeke, 1942**
**Parametric resonance**	**Rajasekar and Sanjuan, 2016**
**Stochastic resonance**	**Rajasekar and Sanjuan, 2016**
**Subharmonic resonance**	**Ludeke, 1942**
**Sympathetic resonance**	**Zhang et al., 2013**
**Vibrational resonance**	**Rajasekar and Sanjuan, 2016**
***Furthermore, as resonance occurs in any physical system with oscillations, there are medium-specific resonances, including the following examples:***	
**Acoustic resonance**	**Ziada and Lafon, 2014**
**Chemical resonance**	**Freeman et al., 2014**
**Electrical resonance**	**Blanchard, 1941**
**Friction resonance**	**Duan et al., 2021**
**Geometrical resonance**	**McMillan and Anderson, 1966**
**Gravitational resonance**	**Baeßler et al., 2015**
**Magnetic resonance**	**Slichter, 2013**
**Mechanical resonance**	**Wilfinger et al., 1968**
**Optical resonance**	**Oldenburg et al., 1998**
**Orbital resonance (mean-motion resonance)**	**Sinclair, 1975; Wang et al., 2021**
**Plasma resonance**	**Dahm et al., 1968**
**Quantum resonance**	**Moran et al., 2017**
**Reaction resonance**	**Yang et al., 2015**
**Tidal resonance**	**Garrett, 1972**
***Additionally, resonances can emerge from the combinations of basic physical forces, such as those illustrated by the following examples:***	
**Electromagnetic resonance**	**Fauché et al., 2017**
**Nuclear magnetic resonance**	**Hore, 2015**
**Plasma-electron resonance**	**Tonks, 1931**
**Spin-mechanical resonance**	**Poshakinskiy and Astakhov, 2019**
**Magneto-mechanical resonance**	**Grimes et al., 2002**
**Electromagnetic acoustic resonance**	**Hirao and Ogi, 1997**
**Nuclear acoustic resonance**	**Sundfors et al., 1983**
**Spin gravitational resonance**	**Quach, 2016**
**Electron spin resonance**	**Wertz, 2012**
**Optical spin resonance**	**Crooker et al., 1997**
***Finally, there are emergent resonances that take on a researcher's name, including the following:***	
**Fano resonance**	**Lassiter et al., 2010**
**Feshbach resonance**	**Tojo et al., 2010**
**Mie resonance**	**Roll and Schweiger, 2000**
**Proudman resonance**	**Vilibić, 2008**
**Schumann resonance**	**Williams, 1992**

Most of the forms of resonance listed in the PHYSICS category appear to be based upon Helmholtz's (2009) idea of sympathetic resonance. For instance, in a review of magnetic resonance, “the term resonance implies that we are in tune with a natural frequency of the magnetic system” (Slichter, 2013). Yet a recent Royal Society review article (Vincent et al., 2021) makes the following claim: **“the definition of resonance has been generalized [to include] all known processes leading to the enhancement, suppression or optimization of a system's response through the variation/perturbation/modulation of any system property.”** This incredibly broad definition of resonance in physics suggests the challenge and need for a coherent understanding of this important concept across physics, neuroscience, the social sciences and design.

This article reviews the role of resonance in human systems, in AI and in human–robot interactions (HRI). Given the general appreciation of resonance in human interactions, we argue that designers can make use of the untapped potential of resonance to shape successful and desirable interactions in AI and HRI.

### Resonance in Human Interactions: A Metaphor, a Mechanism or Both?

“Resonance” is a commonly-used term that describes the human experience of powerful, connecting and activating interactions (Duarte, 2013). For instance, we can “resonate” with a film or with a new friend. Metaphors related to resonance are also common, such as in the expressions “syncing up,” “getting on the same wavelength,” or even “feeling good vibes.”

Although the term “resonance” is often intended as a metaphor to describe an interaction, in many cases physical resonance may also be a mechanism underlying the interaction. For instance, people metaphorically “resonate with music” but the brain also physically resonates with music: the actual frequencies of sound and rhythm can be observed in the frequencies of electrical activity in the brain (Coffey et al., 2019, 2021; Kaneshiro et al., 2021; Pandey et al., 2021). Or, consider the common expression “syncing up” to describe a meeting. Even though “syncing” (that is, synchronization) is intended purely as a figure of speech, human communication does link to measurable “inter-brain synchrony” (Dumas et al., 2010; Dikker et al., 2019; Czeszumski et al., 2020; Kingsbury and Hong, 2020; Dumas and Fairhurst, 2021; Moreau and Dumas, 2021). This article aims to create a bridge between the human experience of resonance and resonance as a physical mechanism. In so far as resonance is more than a metaphor—if resonance is also a causal mechanism in human interactions—then this will have implications for measurement and design.

Some skepticism is justified in viewing resonance in human interactions as “real” rather than as just a metaphor. Historically, sympathetic resonance (sympathy: $\sigma \hat{v}\mu \pi \hat{\alpha }\theta \varepsilon \iota \hat{\alpha }$ or *sumpátheia*) was viewed as the primary mechanism for magical phenomena. For instance, the neoplatonic philosopher Plotinus (205–270) wrote: “But how do magic spells work? By sympathy [sumpatheiai] and by the fact that there is a natural concord [sumphonian] of things that are alike [homoion] and opposition of things that are different.” (Lobis, 2015). Even in the modern era, there remains a widespread belief system that positive thinking or “thought vibrations” can bring about positive real-world occurrences through sympathetic resonance (Atkinson, 1906; Hicks and Hicks, 2006; Ehrenreich, 2009). Perhaps as a result of this association with magic, resonance was not always acceptable as a scientific explanation. A recent column in *Nature Physics* notes:

“…until the very late nineteenth century, scientists were reluctant to use the term ‘resonance' in connection with anything except acoustic phenomena, where it originated. Use of the word in other fields…always included some disclaimer that the link was “only by analogy”, despite the formal equivalence of the fundamental dynamical equations.” (Buchanan, 2019)

Now, the situation has changed: the term “resonance” is abundant in contemporary scientific literature (reviewed in Box 1). However, the term is often used ambiguously, where it is unclear whether “resonance” is being treated as a metaphor or as a physical mechanism. This ambiguity is present in the social sciences as well as in physics. Resonance in physics is an increasingly broad concept that refers to a range of phenomena. To bring clarity, we offer a glossary in Box 2 with proposed definitions for resonance and related terms, such as synchronization, entrainment, reverberation, etc.

Box 2 **Glossary of terms.**

**Resonance**, in this article, is treated as an umbrella term that involves physical resonance (either sympathetic or internal), synchronization, entrainment, memesis and attunement—as well as metaphorical resonance.

**Sympathetic Resonance** occurs when external, forced oscillations are aligned to a system's own natural oscillations and this results in amplification and synchronization. The amplification effect occurs when the natural frequencies of a system align with the frequencies of an external oscillator. Resonance also involves a synchronization or mirroring effect, as the phase and frequency of an external oscillator are reflected in the phase and frequency of the system's response.

**Internal Resonance** involves the activation of the natural frequencies (“eigen frequencies”) of a system. For instance, tapping a wine glass results in internal resonances.

**Synchrony** is a broad term that describes the temporal correlation of independent units of action. Correlations can occur in frequency independently from phase; for instance, heart rate synchrony can occur in people who have the same heart rate, even if their hearts do not beat at exactly the same time. Although all meanings of resonance should refer to a causal phenomena, synchrony can occur without a causal relationship (i.e., correlation does not imply causation).

**Synchronization**, in contrast to synchrony, should be understood as a complex dynamic and causal process—not a state. Pikovsky et al. (2001) carefully define synchronization as “an adjustment of rhythms of oscillating objects due to their weak interaction.” Further, synchronization requires “self-sustained oscillators,” like a powered metronome. Self-sustained oscillators are those that “oscillate with a distinctive waveform at a preferred amplitude that reflects a balance between energy inflow and dissipation.” (Strogatz, 2003). Synchronization also requires a sort of “weakness in coupling.” Weakness is important because a very strong coupling between systems simply results in immediate complete synchronization. The synchronization of two oscillators does not require that the two have the same phase at the same time; for instance, two clock pendulums can be synchronized but swing in anti-phase. When an external oscillation frequency is nearly aligned to a natural frequency of a powered oscillatory system, the systems will “phase lock” together and synchronize. Synchronization can occur at “a rational fraction of the resonance frequency,” like 2:3 or 2:1 (Shim et al., 2007).

**Entrainment** occurs when a consistent rhythmic pulse of one oscillator shifts the frequency of another self-sustained oscillator. For instance, a drummer's beat can entrain the motion of rowers or entrain dancers to a common rhythm. Like synchronization, entrainment requires weakly-coupled and self-sustained (powered) oscillators (Pikovsky et al., 2001). In fact, the two terms are nearly identical; at least one author (Izhikevich, 2007) claims that entrainment is limited to 1:1 synchronization. According to Helfrich et al. (2019), true entrainment requires that “an ongoing oscillator is entrained by a rhythmic input at a slightly different frequency. The entrained oscillation becomes phase-locked and the amplitude increases. After the entraining stream stops the oscillator exhibits a reverberation at the driving frequency for several cycles.” Some definitions of entrainment require that an external oscillator unidirectionally influences a powered oscillator (Lakatos et al., 2019) but other definitions allow for mutual entrainment, “whereby two rhythmic processes interact with each other in such a way that they adjust toward and eventually ‘lock in' to a common phase and/or periodicity” (Clayton et al., 2005). In this article, entrainment is treated as the mechanism for synchronization (synchronization through entrainment) and both terms are treated as types of resonance (see Box 3).

**Reverberation** can be defined by the reverberation time, which is the time required for an oscillation to “fade away” once the external input has stopped. After an impulse, a system's natural or resonance frequencies tend to continue to reverberate, as in a tapped wine glass or echoes in a cathedral.

**Coherence** refers to the statistical similarity between two or more oscillating systems (Wolf, 2010).

**Mimesis**, or imitation, describes the intentional or non-intentional replication of movement patterns. These replications do not need to be synchronous. For instance, a child sticking out their tongue and another child copying them. This can be viewed as a type of resonance enabled by memory.

• Behavioral Mimicry occurs when people behave in similar or identical ways within a short period of time. (Mayo and Gordon, 2020); i.e., “the replication of automated behaviors”
• Imitation can be described as “a short sequence of actions that I see my interaction partner performing and then consequently replicate…imitation is not mere mirroring in the sense that one copies every little part of another's movement. It is rather the replication of the action with regard to the outcome of the action which leads to the acquisition of new skills” (Lorenz et al., 2016).

**Behavioral synchronization** describes behaviors that are synchronous in time, but potentially complementary (e.g., turn-taking) (Chartrand and Van Baaren, 2009).

**Physiological Synchrony** or Biobehavioral synchrony involves the rhythmic and temporal correlation of breath rate, heart rate, hormone production, or interbrain synchrony (Feldman, 2017; Mayo and Gordon, 2020)

**Psychological Attunement** has been defined as “Entrained rhythms [that] constitute a form of dynamic equilibrium in which partners vary their behaviors over time while keeping this variation within desired limits” (Sadler et al., 2009).

**The Vibe** (e.g., “good vibes” or “vibing with”) is a pervasive cultural construct used to describe how people perceive the shared affective experience and aesthetic expectations of a group, a place, a product, a brand, a robot, etc. The vibe is different from a person's individual affective reaction, as it describes the aspects of conscious experience that are perceived to be shared between people (Witek, 2019). Hypothetically, the vibe emerges from interpersonal resonance effects.

**Harmony** is an ancient concept (Lomas et al., 2022) that has an intrinsic relationship with resonance: the harmonic tunings of stringed instruments maximize acoustic resonance between the tuned strings. For example, musical notes with consonant intervals (such as the 2:3 ratio of a musical fifth) will share common acoustic harmonics, while dissonant intervals do not. These shared harmonics produce physical resonances between tuned strings—and perhaps resonances between neural oscillators, as well.

### Resonance as a Physical Mechanism

To provide grounding for resonance in human dynamics, this section outlines physical resonance as a causal mechanism in acoustics. Though we focus on sound, it is important to note that resonance operates in all oscillating systems, regardless of medium. This universality results from the fact that resonance is a mathematical property—it is the natural result of the alignment of phases in oscillating systems.

A wine glass offers an excellent example of the physics of resonance. First, if a glass is gently tapped with a spoon, there will be a *reverberating* sound that reflects the natural frequencies of oscillations in the wine glass. These natural frequencies, which are inherent to the structure of the glass, are also known as characteristic frequencies or eigen frequencies (“eigen” is German for “own” or “inherent”). These natural frequencies are also the *resonance frequencies* of the glass: when external, forced oscillations match these natural frequencies, resonance occurs. But, while tapping the glass with a spoon may reveal the resonant frequencies of the glass, the wine glass is not *in resonance* with the spoon.

**Figure 1:**
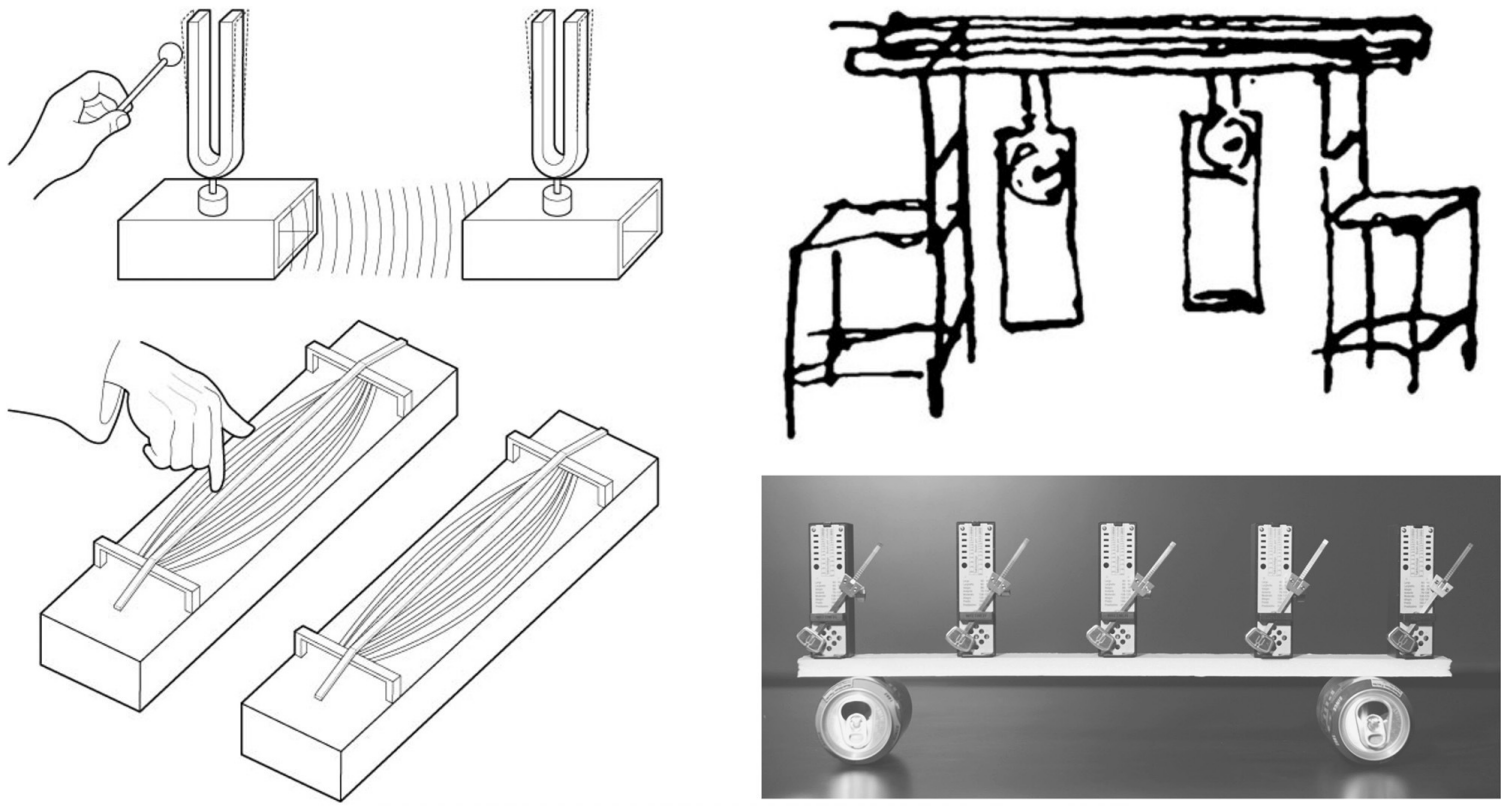
**(Left)** Resonance between mutually attuned tuning forks and strings involves synchronization and amplification. **(Right)** Sketch by Christiaan Huygens (b. 1629), who discovered “the sympathy of two clocks.” When two clocks are placed on a common beam, their two pendulums will eventually synchronize. Right bottom: a set of weakly coupled powered metronomes (self-sustained oscillators) will eventually synchronize. Photo courtesy: Harvard Natural Sciences Lecture Demonstrations.

*Sympathetic resonance* occurs when external, forced oscillations are aligned to a system's own natural oscillations. If a loudspeaker plays the resonant frequencies of a wine glass, the glass will begin to oscillate at much greater amplitudes than if the speaker played other, non-resonant frequencies. Now the glass is in resonance with the speaker. This effect manifests in other common acoustic systems, as well. When one tuning fork is struck near another identical fork, they will both begin to oscillate together, having been coupled together in synchrony *via* the acoustic vibrations. Similarly, two strings tuned to the same note will move one another in synchrony through sympathetic resonance (Figure 1).

The relationship between resonance, synchrony and amplification was articulated by German nineteenth century scientist Hermann Helmholtz. His book “On the Sensations of Tone as a Physiological Basis for the Theory of Music” (Helmholtz, 2009; originally published 1863) offers the first scientific exposition of sympathetic resonance in acoustics. His primary illustration of sympathetic resonance involves the resonance between a church bell and its bellringer. If the bellringer provides consistent pulls at a frequency that aligns to the bell's natural rate of swinging, then the swinging will be rapidly amplified. Importantly, the sympathetic resonance occurs when phases of oscillation align: that is, when the downward pull of the bellringer matches up with the downward motion of the bell's swing. The role of synchrony in sympathetic resonance is easier to observe with a slow bell ringer than with the rapid oscillations of a wine glass. Yet, even the sympathetic, synchronized oscillations of a wine glass can be made visible with high frequency camera equipment (Slow Mo Guys, 2021).

Synchrony between systems does not necessarily imply sympathetic resonance. Two systems might be synchronized with each other due to a third system, for instance, or for other non-causal reasons (Hasson and Frith, 2016). Other forms of resonance only occur with powered oscillators (like the clocks and metronomes in Figure 1), namely entrainment and synchronization. These terms—which explain phenomena like the synchronization of fireflies or the entrainment of dancers to a musical beat— are defined and discussed in Box 3. In this article, we treat these two terms as subsets of sympathetic resonance (by analogy, like squares are subsets of rectangles).

Box 3 **Clarifying the relationship between resonance, entrainment and synchronization.**

This article treats resonance as an umbrella concept that includes both metaphorical resonance and resonance as a physical mechanism. As resonance has an expansive meaning in physics (Box 1), we first distinguish between typical resonance (which involves the natural frequencies of a system) and atypical resonance (which does not involve natural frequencies). Then, typical resonance includes *internal resonance* (the activation of reverberating natural frequencies) and *sympathetic resonance* (alignment between the frequencies of external oscillations and the natural frequencies of a system). Sympathetic resonance can be further divided into *passive resonance* and *active resonance*. Passive resonance occurs with unpowered oscillators (like a wine glass) while active resonance occurs with powered or *self-sustained oscillators* (like battery-powered metronomes). Active resonance includes the phenomenon of *entrainment*, which occurs when a self-sustained oscillator synchronizes its phase and frequency to a weakly coupled external oscillation.

The natural or resonance frequency of an oscillator can be considered its *preferred frequency*. Powered oscillators, like metronomes, also have a *preferred amplitude* of oscillation—this is not the case for passive oscillators, like wine glasses. When external frequencies align with the preferred frequencies of a wine glass, the most noticeable aspect is the amplification of the amplitude of the oscillation. In contrast, when external oscillations align with the natural frequencies of a powered oscillator, the most noticeable aspect is the synchronization. However, the synchronization of frequency and phase also occurs between a wine glass and an external speaker while amplification also occurs with synchronized metronomes. For this reason, we describe entrainment and synchronization as types of sympathetic resonance; namely, the type involving a self-sustained oscillator.

The scientific relationship between entrainment and resonance is often a point of confusion due to the lack of clear definitions (Helfrich et al., 2019). Our view diverges from other descriptions of resonance that are limited to passive, unpowered systems (Pikovsky et al., 2001; Guevara Erra et al., 2017; Lakatos et al., 2019). Our view is that the concept of resonance can easily accommodate active forms as well as passive forms, as both involve preferred frequencies of oscillation (natural frequencies), synchronization effects and amplification effects. Given the ubiquity of resonance in oscillatory systems—and its already expansive definition in physics (Box 1)—why should resonance only refer to unpowered systems and thus exclude dissipative systems, like the brain? Rather than treating “resonance” in interpersonal interactions as a complete misnomer, we make the case that it is appropriate and physically accurate to say that we *resonate* with people, films or other media. We hope that this view opens the door to a more comprehensible and coherent scientific study of resonances in human interactions.

**Types of Resonance**

• Metaphorical Resonance
• Physical Resonance
  ∘ Atypical Resonance: Does not involve natural frequencies (see Vincent et al., 2021)
  ∘ Typical Resonance: Involves natural frequencies
    ■ Internal Resonance (involves the activation and reverberation of natural frequencies within a stimulated system, like the reverberations of a tapped wine glass)
    ■ Sympathetic Resonance (involves the alignment of external frequencies with the natural frequencies of a system—when the forced frequencies match the natural frequencies)
      • Passive Resonance: unpowered and externally sustained; like a wine glass vibrating in synchrony with external oscillations.
      • Active Resonance: powered and self-sustained, like a metronome synchronizing with external oscillations. This encompasses different types of synchronization:
        ∘ Complete Synchronization (due to strong coupling)
        ∘ Entrainment (Phase Synchronization)
          ■ In-phase
          ■ Anti-phase
          ■ Phase shifted
        ∘ Frequency Synchronization
        ∘ Envelope Synchronization
        ∘ Partial and Asynchronous Synchronization (e.g., mimesis)

### Resonance as a Metaphor

Having briefly considered the operation of resonance as a physical mechanism, we now wish to bring clarity to the metaphorical use of resonance in science and broader culture.

A recent review of the word “resonance” in the language of scientific literature (Ruthven, 2021) reveals that resonance typically serves as an implicit metaphor to indicate 1. agreement (e.g., new evidence can resonate with an existing theory), 2. arousal (e.g., a film that resonates is engaging and moving) or 3. action (e.g., the resonance of a speech can motivate people to take action). But, despite a vast number of scientific articles that use resonance as a term, it is only very rarely defined. The lack of definition suggests that “resonance,” as a term, is easily and broadly understood *intuitively* as a metaphor.

Metaphors are useful when they enable concrete, familiar experiences to communicate abstract, conceptual meanings (Lakoff and Johnson, 1980; Yang, 2014). A metaphor involves the pairing or alignment of concepts between a concrete source and a more abstract target; this coupling of concepts produces mappings that allow multiple concepts to be integrated together into an emergent space of meaning (Holyoak and Stamenković, 2018). An example set of metaphorical mappings use the metaphor “Love is a Journey”: for instance, in a journey (the source) there are travelers while in love (the target) there are lovers. In Table 1, we provide a similar set of explicit mappings between the metaphor of acoustic resonance and resonant human interactions.

**Table 1:** A hypothetical conceptual mapping of acoustic resonance and human resonance.

**Acoustic resonance (source)**	**Human resonance (target)**
A string tuned to another string can respond sympathetically	A person attuned to another person can respond sympathetically
A string's sympathetic response to another string results in synchrony	A person's sympathetic response to another person results in synchrony
A string mirrors the vibrations of another string	A person mirrors “the vibe” of another person (see Box 2)
A string vibrates to the frequency of another string, if tuned	A person responds to the expression of another person, if attuned
The attunement of strings is based on a common or an aligned set of oscillations	The attunement of people is based on a common background or an aligned set of experiences
Tuning strings enhances the resonance between strings	Attuning people (e.g., with common experiences) enhance resonance
Resonance results in greater amplitude of sound	Resonance results in greater excitement in people
A string will only selectively resonate to particular frequencies, based on its own natural oscillations	A person will only selectively resonate to particular [people, films, books, etc], based on their own natural propensities

With this introduction to resonance established, we are now posed to explore the alignment between the metaphorical experience of resonance (as in a film that resonates) and the physical phenomena of resonance itself. The next section of the article considers the research basis for understanding resonance in human interactions.

## Part 1: Resonance in Human Interactions

A common example of physical resonance in human interactions can be found on nearly any playground. When pushing someone on a swing, the pusher needs to coordinate the timing of their pushes to the swing's natural back-and-forth oscillation (determined primarily by the length of the swing). Does pushing at a faster rate help? No: if the pusher simply pushed more times per second, most of the pushes would do nothing because they would not line up with the movement of the swing. When a pusher aligns their timing to the natural frequency of the swing, they amplify the effects of their effort: many small, well-timed pushes are enough to get the swinger high into the air.

Beyond this simple example, where else might resonance occur in human interactions? To scope our search, we assume that sympathetic resonance can only occur when external oscillations and natural oscillations align. Therefore, physical resonances in human systems should only be present during human activities that have a natural frequency or rhythm of oscillation.

Consider an everyday rhythmic human activity: walking. Researchers have used accelerometers to determine the dominant and natural up-and-down frequency of walking. The typical frequency of naturalistic walking is about 2 Hz, or two steps per second (MacDougall and Moore, 2005). This natural oscillatory frequency can vary—some people walk faster or slower than others. However, across a diverse set of participants, the researchers found that the tempo of walking was not dependent upon height, weight, or other physical factors. In fact, the researchers suggest that the 2 Hz natural tempo is the result of genetically encoded “central pattern generators” in the spinal cord, as these are the basis of the tempo of locomotion in other animals (Guertin, 2013).

This is not just trivia: structural engineers need to take into account this 2 Hz human walking pace in every footbridge that is built. Famously, a 2 Hz resonance frequency caused the UK's Millenium Bridge to dramatically sway side-to-side when it was loaded with pedestrians (Dallard et al., 2001; Strogatz et al., 2005). On opening day, the bridge became so crowded that most people could not easily walk forward—instead people were so packed-in that they had to walk in a sort of side-to-side waddle. Unfortunately, the bridge had a natural side-to-side 2 Hz resonant frequency. As the bridge started to sway back and forth, this led to the synchronization of the waddling motion of the thousands of pedestrians. Without deliberate coordination, people stepped left and stepped right in synchrony with each other, entrained to the swaying motion of the bridge. As a result, the 2 Hz side-to-side oscillation of the bridge was further amplified to dangerous levels.

**Figure 2:**
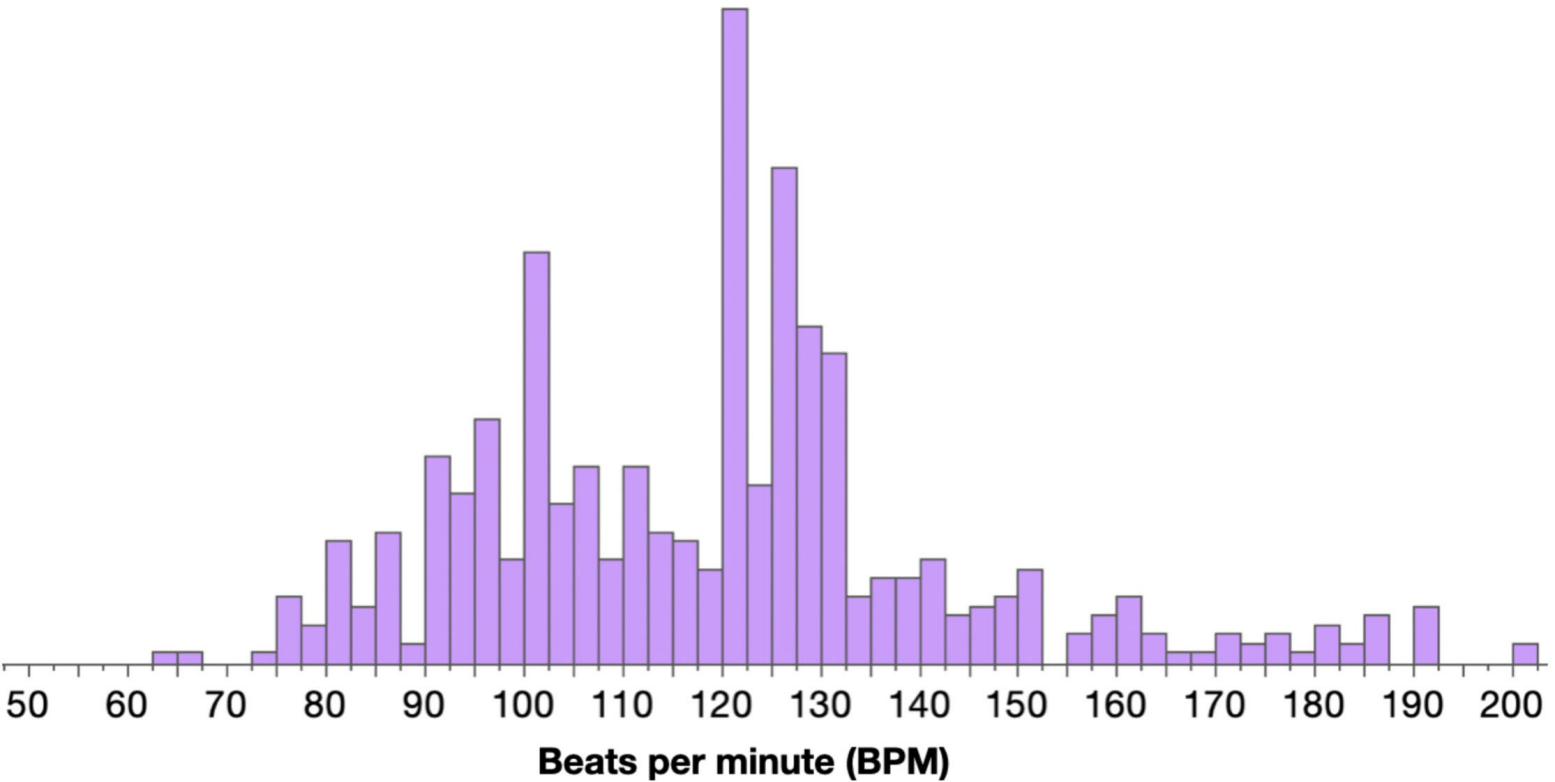
The above histogram is compiled from a Kaggle dataset containing the “Top 50” Billboard songs from 2010 to 2019. It appears to be a resonance curve showing maximum excitement at a preferred frequency of oscillation. However, note the sharp dropoff from 120 BPM to 118 BPM—this is not expected in a resonance curve, as the frequencies close to resonance tend to resonate strongly as well. There may simply be very few songs released with this BPM. But, while this graph might not be a resonance curve, it may be a result of meaningful resonance effects in the brain—an increase in amplitude due to the alignment of external frequencies with natural frequencies https://www.kaggle.com/leonardopena/top-spotify-songs-from-20102019-by-year.

Given that 2 Hz is a *natural* frequency of human movement, the theory of resonance predicts that 2 Hz should also be a resonance frequency. That is, external rhythmic inputs at about 2 Hz should cause a synchronization and amplification of human movement. Is 2 Hz actually a resonance frequency of movement? One way to test this prediction is to consider the popularity of music at different beats per minute (BPM), where 120 BPM would be equivalent to 2 Hz. Figure 2 presents a histogram showing the relative distribution of BPM in the Top 50 songs for each year of the past decade, worldwide. This reveals a distinct peak at 120 beats per minute.

Is this a resonance effect? The graph in Figure 2 resembles a resonance curve: given a range of input frequencies, there is a selective amplification at the same frequencies as the natural frequencies of the stimulated system (i.e., the 2 Hz natural pace of movement in humans). However, the peak may arise for a variety of reasons, from the musicians' recognition of the popularity of 120 BPM to the listeners' increased familiarity with 120 BPM songs. Most importantly, there are many more songs released at 120 BPM. So, even though it is not entirely appropriate to refer to the graph as a resonance curve, it may still be a function of resonance effects (e.g., a greater likelihood of rhythmic entrainment to walking frequencies at 120 BPM).

In this article, we propose that physical resonance can be distinguished from purely metaphorical resonance when the mathematics of resonance (as used to characterize other physical systems) can be used to model human interactions. Synchronization, amplification and signal alignment are the mathematical hallmarks of resonance—therefore, physical resonance in human systems should occur when the oscillations of external signals match natural human oscillations and this results in synchronization and increased energy (amplification). This viewpoint treats the physics of human resonance in a similar fashion as other physical systems yet it leaves room for future research to further clarify the relationships involved.

### Rhythmic Human Interactions

The previous example shows how rhythmic human activity can be investigated as a context for physical resonance phenomena. Rhythmic human interactions clearly occur in artistic domains, such as music-making (Clayton et al., 2005), dance (Larsson et al., 2019) and various kinds of cultural rituals like chanting (Gelfand et al., 2020). Rhythmic interactions are also common in everyday life, as in the case of walking, conversational turn-taking (McGarva and Warner, 2003; Wilson and Wilson, 2005; Lee et al., 2010), patterns of eye contact (Wohltjen and Wheatley, 2021) or with interactions like handshakes (Melnyk and Hénaff, 2019). More intimate rhythmic interactions occur during human sexual behavior (Safron, 2016). Leading up to the moment of birth, midwives often advise expectant mothers to push in phase synchrony with their own rhythmic uterine contractions (Hanson, 2009). Researchers have also observed that the earliest interactions between parent and child are strongly rhythmic (Stern et al., 1985). Babies cry in a rhythmic manner and caregivers soothe them with synchronized motions (Trehub and Trainor, 1998). Synchrony in caregiving appears to literally “tune” the human social brain (Yaniv et al., 2021).

“This resonance or echoing of affect, feelings, and emotions that takes place in the reciprocal interaction between infants and their caretakers is a necessary element for the development of empathy and advanced social cognition.” (Decety and Meyer, 2008)

### Neurobiological Rhythms and Human Nature

What makes human behavior so rhythmic? Human rhythms are believed to emerge from a broad range of biological oscillators that are present across the brain and the rest of the body (Varga and Heck, 2017). For instance, rhythmic central pattern generators in the spinal cord not only drive locomotion (Ijspeert, 2008; Guertin, 2013), but also drive heart rhythms (Bucher et al., 2015) and breath rhythms (Molkov et al., 2014). Furthermore, body and brain oscillations appear to integrate together in a hierarchical architecture (Klimesch, 2018).

Rhythmic—and resonant—phenomena are found in the brain at multiple levels, from neurons to circuits to brain waves (Buzsaki, 2006). Individual neurons have natural intrinsic oscillatory periods (Lampl and Yarom, 1997); neurons can be *tuned* to respond to different input frequencies through resonance, like “strings on a violin” (Das et al., 2017). At the level of neural circuits, the *reverberation* of recurrent activity in neural ensembles plays a key role in memory (Wang, 2001; Tegnér et al., 2002; Han et al., 2008), as originally predicted by neuroscientist D.O. Hebb in 1949 (Hebb, 2005). Finally, large-scale electrical oscillations in the brain, or brainwaves, demonstrate clear resonance effects (Herrmann, 2001) that are observable through electroencephalography (EEG). Further to this, neurobiological processes associated with adaptive learning (Grossberg, 2013, 2017), perceptual learning (Raja, 2020), and ecological cognitive architecture more generally (Raja, 2018), have all been theorized as forms of physical resonance.

Neurons are, technically speaking, non-linear oscillators (Izhikevich, 2007; Stiefel and Ermentrout, 2016)—therefore, it is not so surprising that large collections of neurons exhibit both internal resonance effects (one part of the brain resonating to another) and external resonance effects (the brain resonating to environmental phenomena). Many scientists believe that physical resonance in the brain plays a major role in music perception, such as the physicist and neuroscientist Ed Large, who claims:

“The brain does not ‘solve' problems of missing fundamentals, it does not ‘compute' keys of melodic sequences, and it does not “infer” meters of rhythmic input. Rather, it resonates to music… certain aspects of this process can be described with concepts that are already well-developed in neurodynamics, including oscillation of neural populations, rhythmic bursting, and neural synchrony.” (Large, 2010)

Some researchers have recently proposed a unified account of rhythmic synchronization and entrainment in the brain (Lakatos et al., 2019); other researchers have proposed a unified account of the biological, neurological and physical mechanisms involved in the “rhythmic entrainment of biological systems” (Damm et al., 2020). Rhythmic entrainment has been found to govern patterns of interaction at a social, population, and even species level—where, in the latter case, the entrainment of natural oscillations can be observed at the scale of economies and ecosystems (Greenfield et al., 2021). In short, it would appear that resonance effects can operate all the way up and all the way down: from neurons to economies.

### Entrainment and Rhythmic Synchronization

Human interactions can naturally synchronize through the process of entrainment (Boxes 2, 3), which is akin to the natural synchronization of metronomes (Figure 1). Social neuroscientist Ruth Feldman (2012, 2017) argues that biobehavioral synchrony (in behavior, heart rate, endocrine production and brainwaves) serves as a key principle underlying parental love, romantic love, friendship and human attachments. Indeed, when loving human partners interact, their rhythmic communication produces measurable physical synchronization in behavior (Grafsgaard et al., 2018), in heart rate (Prochazkova et al., 2022) and in the brain (Kinreich et al., 2017).

Some argue that the ability to synchronize to a beat is one of the core skills associated with human social behavior. Kirschner and Tomasello (2009) found that children 2–4 years old could adjust their natural drumming tempo to match another beat—but that their accuracy in synchronizing was significantly higher when they drummed with a human partner (as opposed to drumming along with a machine or drumming along with a drum sound produced by a speaker). The authors argue that “drumming together with a social partner creates a shared representation of the joint action task and/or elicits a specific human motivation to synchronize movements during joint rhythmic activity.”

Humans are typically much less able to synchronize to rhythms of visual flashes than to rhythms of auditory tones (see review by Repp and Su, 2013). But, rhythmic entrainment and synchronization is not specific to music or auditory experiences. With certain forms of visual stimuli (i.e., bouncing balls), visual synchronization becomes nearly as accurate as auditory synchronization (Iversen et al., 2015). Researchers have also found that deaf individuals exhibit enhanced synchronization to visual rhythms, suggesting that the ability to attune to rhythms is at least partially based on experience and not just a result of biological coupling between the auditory and motor system (Iversen et al., 2015). Researchers have found that humans can synchronize to tactile pulses on their back with higher accuracy when feeling the vibrations played over their entire back rather than at just a small portion; similarly, rhythms that engage multiple sensory modalities also produce more accurate synchronization (Ammirante et al., 2016). This suggests that overall sensory immersion and attentional engagement affects the propensity to synchronize with rhythms.

Synchronization helps support coordinated actions between individuals. Meta analyses (Morgan et al., 2017) have shown that behavioral synchrony in groups increases prosocial behavior, increases perceived social bonding, and generally *feels* good (as measured as increases in positive affect). Why might behavioral synchrony feel good? Cracco et al. (2021) claim that “synchrony is aesthetically pleasing and a signal of group cohesion, as stimuli that are processed more fluently are known to produce a hedonic response.”

Rhythmic synchronization is very rare in the animal kingdom, at a social level, apart from special examples (see extended discussion in Box 4). The human capacity for rhythmic synchronization may have coevolved in human cultures because it enhanced social bonding between sexual partners, between parents and children, and within larger social groups. Savage et al. (2021) state:

“‘Neural resonance' (synchronous brain activity across individuals) facilitates social bonding through shared experience, joint intentionality, and ‘self-other merging'. Through the production of oxytocin and endogenous opioids, neural resonance also facilitates prosociality.”

Box 4 **Resonance in non-human animals.**

Despite the relative utility of resonant phenomena in humans (such as social synchronization and entrainment), it is rare in the animal kingdom. There are examples of animals synchronizing with other members of their species: for instance, chirping insects, croaking frogs, claw-waving crabs, and flashing bioluminescent animals, like fireflies (Wilson and Cook, 2016). However, these examples seem to involve somewhat involuntary neurological connections in fairly simple animals. Why is entrainment not more common? While this is not well-understood at present, Wilson and Cook (2016) provide three criteria for rhythmic entrainment: 1. an animal needs to have the mechanical ability to move with the beat (i.e., the tempo should be similar to an animal's natural tempo of movement), 2. the animal must be able to extract the beat from the sensory environment and pay attention to it, and, crucially, 3. the animal must have the *motivation* to voluntarily move in union.

Until very recently, it was believed that only human beings could synchronize to an external rhythm, like a musical beat. It was only with the advent of YouTube that researchers first discovered Snowball the Dancing Cockatoo (Patel et al., 2009; Patel and Iversen, 2014) and 33 other examples of animals that appeared to show entrainment to music (Schachner et al., 2009). These examples spanned 14 different bird species—and an Asian Elephant. This led to the belief that only species that had previously evolved the capacity for vocal mimicy could entrain to a beat. Schachner et al. (2009) noted that, despite a large number of “dancing” dog videos, none demonstrated the ability to synchronize with music (even though some dogs had been trained for years to compete in dance competitions).

Some animals can be reliably trained to synchronize to a rhythm. In 2011, researchers demonstrated that Budgerigars, a parrot-like bird, could learn to produce rhythmic beak tapping patterns that synchronized to an audio-visual metronome (Hasegawa et al., 2011). Then researchers managed to train Ronan, a Sea Lion (Cook et al., 2013), to entrain to a beat—this was surprising because Sea Lions are not vocal learners.

Over the past decade, there has been much investigation of the capacity for non-human primates to entrain to a musical beat. Sounds can induce spontaneous rhythmic swaying in chimpanzees (Hattori and Tomonaga, 2020)—however, this swaying effect occurs in response to randomized rhythms and when sounds are rhythmic (Bertolo et al., 2021). Monkeys have been trained to tap in response to an auditory or visual metronome, however, their movements are always reactive: they always tap following the stimuli (although much faster than they can in a single reaction time experiment; see Wilson and Cook, 2016). In contrast, when humans entrain to a similar metronome, they typically tap slightly *before* each stimulus in the beat. In just one case, researchers have trained monkeys to make predictive, synchronized eye movements to a visual metronome—however, the monkeys had to be rewarded for each trial (Takeya et al., 2017). Based on this evidence, the authors suggest that monkeys and other animals may have the capacity for “predictive and tempo-flexible synchronization to a beat” but might not be “intrinsically motivated” to synchronize!

In summary, it is surprising that so few animals—neither dogs nor monkeys—are predisposed to entrain to a beat. After all, even animal *neurons* have the capability to entrain to periodic rhythms. Why, then, are animals generally so unable—or unwilling— to entrain to a beat? One possibility: consider that the heart is entrained to rhythms produced by central pattern generators in the spinal cord; clearly, animals need to protect their heartbeat from becoming entrained to external stimuli. It may be that, even in the simplest of animals, there is a need to evolve *defense mechanisms* that can protect against unwanted resonance effects. Part of the human capacity for rhythmic entrainment may result from the ability to “let one's guard down” in order to open up to certain kinds of external rhythmic entrainment with other people. This would suggest that humans only resonate to external stimuli when they feel safe to do so; after all, stress may make it difficult to dance or to be moved by music. This also suggests that animals may be able to resonate, if they could be emotionally or biochemically prepared to do so. This opens up possibilities for animal-robot and animal-AI interactions that can be explored in the future.

### Hyperscanning and Inter-brain Synchrony

The scientific understanding of rhythmic entrainment and neural resonance is a fast-moving area of neuroscience that is being propelled by new hyperscanning methods that scan the brains of multiple interacting participants simultaneously. Interpersonal neural synchrony at the group and dyadic level has been shown to be associated with a number of predictors, including shared stimulus features, joint actions, personality traits, social intentionality, relationship quality, and cooperation (see, e.g., Czeszumski et al., 2020 for review).

For example, recent work from co-authors of this article investigated the relationship between inter-brain synchrony and group dynamics and found that EEG inter-brain synchrony predicted collective performance among teams better than self-report (Reinero et al., 2021). In another line of work, group-based inter-brain coherence predicted class engagement and social dynamics in groups of high school students during their real-world lessons (Dikker et al., 2017; Bevilacqua et al., 2019). Social closeness with the teacher also correlated with brain-to-brain synchrony—that is, enhanced synchrony was found with students who reported greater engagement with the teacher. Finally, and perhaps most directly related to the concept of resonance: Brainwaves of students who engaged in face-to-face interactions *before* class were more synchronized *during* class, even if students were no longer interacting. This finding raises interesting questions about the role of resonance in the directionality of the relationship between human face-to-face interaction, inter-brain synchrony, and social connectedness.

### The Downside of Being in Sync: Chained to the Rhythm?

Humans may be predisposed to synchronize with each other, but this does not always lead to positive outcomes. Synchronization also has some important tradeoffs; Gelfand et al. (2020) claim that synchrony can produce conformity, destructive obedience, groupthink, antisocial aggression and also impair group creativity. They point to findings (Wiltermuth, 2012a,b) that people who have been randomly assigned to a synchronous activity are more likely to comply with an anti-social order (e.g., irritating a stranger) and to follow a morally compromised command (in the study, participants were asked to grind up live bugs). Synchrony also increases the likelihood that people will engage in conformity, like copying majority opinions rather than following their personal preferences (Dong et al., 2015). Further, sometimes synchrony is simply “situationally inappropriate;” in a study of a complex verbal coordination, groups that were randomly assigned to a synchronization task performed worse, reported higher levels of conflict and reduced group cohesion (Wood et al., 2018).

Gelfand et al. (2020) randomly assigned participants to march synchronously around a college campus or at their own pace. The participants who synchronized showed reduced creativity when writing stories. They also found that synchronous marching discouraged the development and sharing of minority perspectives during decision-making. They relate this finding about synchrony to the need to balance “tightness” and “looseness” in culture.

The ability to flexibly move in and out of synchrony appears to be critical to adaptive flexibility. Mayo and Gordon (2020) claim that “two tendencies exist simultaneously, one to synchronize with others and another to move out of synchrony and act independently. We suggest that an adaptive interpersonal system is a flexible one, able to continuously adjust itself to the social context.”

Savage et al. (2021) point out the key difference between rhythmic integration and pure synchronization: rhythm is predictable but also flexible to accommodate diverse individual contributions. This is because rhythm involves two essential components: 1. equally timed beats (isochronicity) and 2. a hierarchical structure (meter). “While synchronization solely to the beat (e.g., in marching or unison chanting) allows large groups to integrate, it tends to submerge individual contributions. Meter solves this problem by allowing many individuals to contribute, out of phase, to the same integrated rhythm.” Social rhythms (of speech, music, dance, etc.) can thus support diversely coordinated actions within a loosely unified structure.

### Origins of Empathy: Sympathetic Resonance

Sympathetic resonance—including synchronization and rhythmic entrainment—appears to have been a key factor in human evolution (Savage et al., 2021; Lin and Lomas, 2022). Resonance relates in a fundamental way to the human capacity *to feel what another person feels*, which is often called empathy. But, before the term “empathy” was coined in the twentieth century, the ability to feel what others feel was referred to as “sympathy”—as in sympathetic resonance. The eighteenth century philosopher Adam Smith wrote his first book, “The Theory of Moral Sentiments” (Smith, 1759), with the general thesis that “sympathy” accounts for a large portion of moral behavior. Specifically, he explained that people like to help other people because they sympathetically feel good when other people feel good and sympathetically feel bad when other people feel bad (Schliesser, 2015).

Later, the nineteenth century German psychologist Theodore Lipps used the German term *Einfühlung* to describe how people “feel into” the states of other people and even art pieces. By using an inner imitation or simulation, people seem to be able to fuse with artworks or persons through a process of “Psychische Resonanz” (Lipps, 1891). For instance, watching a tightrope walker produces a resonance with internal associated feelings like vertigo. The representation of the performer in one's own mind allows one to feel how oneself would feel in the same situation. The psychologist Edward Titchener reviewed Lipps' work in 1909 (Titchener, 1909) and, rather than using the German *Einfühlung*, he coined the new English word *Empathy* (Schliesser, 2015).

### Empathy, Motor Resonance and “Mirror Neurons”

Empathy is viewed as a critical component of human social interactions. However, it is extremely challenging to pin down. While there is an enormous amount of scientific work on empathy, there is still considerable debate about its definition (Hall and Schwartz, 2019). Is empathy a singular capability or does it result from a “laundry list” of characteristics? Psychologists generally accept the division between *cognitive empathy* and *affective empathy*. Cognitive empathy refers to the ability to recognize and understand another person's mental state (cognitive processes captured by what is referred to as “theory of mind” or mentalizing), while affective empathy refers “the ability to vicariously experience the emotional experience of others” (Reniers et al., 2011). Furthermore, psychologists will often draw another distinction between empathy (which involves the ability to distinguish the experience of another person's emotion from one's own emotional state) and *emotional contagion*. Emotional contagion involves the direct propagation of emotional states; unlike empathy, this effect is common in non-humans, like mice (Hernandez-Lallement et al., 2020).

Regardless of definition, the capacity for empathy (or, at least, affective empathy) is typically conceptualized as emerging from *motor resonance*. Motor resonance describes how the spatial-temporal activations of an observer's brain mirrors the brain activations of another person as they perform some set of actions. That is, when observing the physical behavior of another person, the brain regions related to this behavior activate in both the observer and the person enacting the behavior, creating a sort of spatial-temporal synchrony between observers and actors. Thus, motor resonance is a type of physical resonance that provides a mechanism for sharing conscious experiences between people. For instance:

“…the coupling between action and perception, also named “motor resonance” [involves] the automatic activation, during actions perception, of the perceiver's motor system. During action observation the two motor brains “resonate” because they share a similar motor repertoire”. (Sciutti and Sandini, 2017)

Several theories of empathy describe motor resonance as the mechanism underpinning the *mirroring processes* of emotions and actions, where mirroring processes are defined as automatic processes for internal imitation (Iacoboni, 2009) or embodied simulation (Gallese, 2009). The simulation theory of empathy (e.g., Preston and De Waal, 2002) suggests that people can feel what other people are feeling because observing another person's behaviors will coactivate or call upon neural representations of one's own bodily experience (Hurley, 2008).

While stereotypical expressions may produce meaning symbolically (e.g., a smile is symbolically associated with joy or a frown with sadness), human emotional expression is far more dynamic, expressive, and context dependent. The spatial-temporal dynamics of, say, a rapidly lifted eyebrow can express inner emotional states with great specificity. Observing the eyebrow rapidly lifting will engage our own motor cortex to activate in a similar time scale and specifically in the areas expected for the muscles involved. These spatial-temporal activations appear capable of automatically triggering associated emotional states (Wood et al., 2016). That is, whatever feelings might have been associated with rapid eyebrow lifting in the past, either in the self or in others, will now be primed. In this manner, interpersonal motor resonance appears to support “mind reading” (Agnew et al., 2007) and the sharing of conscious experiences (Lin and Lomas, 2022). Because our brains reflect or mirror each other, through resonance, we can sympathetically experience the feelings associated with other's actions, in part by knowing “how we would feel if we were acting that way.” And it is not just observation: even listening to descriptions of actions can trigger motor resonance (Zwaan and Taylor, 2006).

Researchers continue to debate the origins of mirroring processes, but they appear to result from simple bidirectional associations between perception and motor responses that are learned over time (Keysers and Gazzola, 2009, 2014; Hanuschkin et al., 2013). Simple correlations of associated actions and observations seem to produce “action perception circuits” that serve as the neural mechanism for mirroring processes (Pulvermüller, 2018).

For a clear example, fMRI results show that when people watch others perform actions with their hands, mouths or feet, there are activations in their own premotor cortex—activations that are also triggered when performing those actions themselves. Furthermore, these action activations occur “following a somatotopic pattern which resembles the classical motor cortex homunculus.” (Buccino et al., 2004a) For instance, if person A watches person B kick a ball, the “leg part” of the premotor cortex will show a similar pattern of activation in person B (the kicker) and in person A (the observer).

### The “Like Me” Hypothesis

If we consider the sympathetic resonance of two tuned strings, the strings have in common their natural frequencies of oscillation. A similar sympathetic resonance occurs when we see another person smile; this can trigger similar action representations in our brain and activate associated emotional states. Following the metaphor of two tuned strings, the “like me” hypothesis predicts that the degree of motor resonance between an actor and observer will correlate with the degree of *similarity* between the actor and the observer.

Buccino et al. (2004b) investigated human motor resonance in response to dogs, monkeys and people. They found that “Actions belonging to the motor repertoire of the observer (e.g., biting and speech reading) are mapped on the observer's motor system. Actions that do not belong to this repertoire (e.g., barking) are essentially recognized based on their visual properties.” In other words, actions that are not “like me” may be recognized but they do not resonate.

If resonance is enhanced when observing actors similar to the observer, is it also impaired when there is a lack of similarity? Researchers have found that when subjects observe people of a different ethnicity, there is significantly less motor resonance than when watching members of the same ethnicity (Gutsell and Inzlicht, 2010; Azevedo et al., 2013). This unfortunate effect is predicted by resonance theory: less similarity, less resonance.

The resonance between actions is dependent upon a person's ability to do those actions. The “like me” hypothesis predicts that persons who are highly trained in a particular skill should be able to resonate with another person trained in the skill, at least to the extent that their action-observation circuits are mutually developed. Work with expert dancers using fMRI (Calvo-Merino et al., 2005; Cross et al., 2006), EEG (Orgs et al., 2008), and facial EMG (Kirsch et al., 2016a) provides evidence in support of this idea that shared learning experiences and shared skills will increase motor resonance.

Evidence against the Like-Me hypothesis comes from the fact that similarity does not always enhance activation intensity. A series of fMRI experiments showed that mirroring processes (also known as the “Action Observation Network” or AON) are more strongly engaged during the observation of *robot-like* motions, both when the motions were performed by actual robots and when people act in a jerky, robot-like manner (Cross et al., 2012). It appears that the relationship between familiarity and neural resonance is not entirely linear because—in part—the perception of novelty (Knight and Nakada, 1998) also amplifies the brain's response to actions (Gardner et al., 2017).

The brain's sensitivity to novelty may help explain why motor resonance is exceptionally amplified when expert dancers observe other expert dancers perform (Cross and Ramsey, 2021). Experts not only have deep familiarity with the movements but they will also have an expert sensitivity to the many small novelties within the expert's individual execution. Familiarity and novelty—though seemingly opposite—both contribute strongly to an aesthetic experience (Hekkert et al., 2003). This may account for why aesthetically valued actions influence motor resonance. Researchers have found that the intensity of activations in the AON (a brain marker of motor resonance) correlates with aesthetic ratings of the observed dances (Cross et al., 2011). Calvo-Merin et al. (2008) also examined the neural response to dance movements and noted that, of five aesthetic dimensions (like-dislike, simple-complex, dull-interesting, tense-relaxed and weak-powerful), only shifts in liking-disliking correlated with the brain response in the AON.

Thus, several factors can enhance motor resonance, including inter-subject similarity. Generally speaking, motor resonance appears to be enhanced by the overall motivational relevance of the subject-observer interaction; the similarity of the subject, the novelty of the interaction, the aesthetic quality of the interaction, and when the subject is viewed as desirable, powerful or sharing a common goal (Greenberg, 2019). This also implies some possibilities for breakdown and pathologies in psychological relationships due to *misattunement* in interpersonal resonance (Bolis et al., 2017).

### Human Resonance, Exemplified by the Actor Will Smith

To conclude this section on the role of resonance in human interactions, consider this quote by the actor Will Smith explaining his growth as an actor during his performance as Richard Williams, the father of Venus and Serena Williams. The quote illustrates the connection between the body, the communication of emotional depth and how resonance is generated during aesthetic experiences (described here as “vibrations”). It also shows the vast distance roboticists must travel to approach the capabilities of human actors in producing effective sympathetic resonance (for resonance in acting, also see Bogart, 2021).

“At the core, acting is what you can comprehend emotionally. And when you comprehend it emotionally, do you understand it enough to feel it and create interesting behavior around it? So something like Richard Williams's walk: Now, you can mimic someone's walk and look authentic. It's a completely different thing when you know why the person is hunching over vs. the stand-up-comedian version of it just mimicking it. Understanding that was the leap that happened: When you know why Richard Williams's left leg hurts, what happened with the spike that got driven through it, that, as an actor, is the 90 percent of the iceberg that's below the surface. When you've programmed it deeply, those things have corresponding vibrations for the audience that they don't even realize.” (New York Times, 2021).

## Part 2: Resonance in Robotics

In this article, we use “robot” as a general shorthand for a non-human artificial agent. This deliberately broad definition includes many forms of intelligent and autonomous systems that vary in the degree of adaptivity (from highly adaptive to non-adaptive, e.g., a pre-programmed movement sequence), in the degree of embodiment (from physical to virtual), in the degree of human resemblance (humanoid to non-humanoid), in the degree of biological resemblance (highly life-like to highly machine-like) and in the degree of social interactivity (highly social to non-social). A typical chatbot, for instance, is a moderately adaptive, virtual, humanoid, machine-like and highly social robot. In contrast, a Roomba is a highly adaptive, embodied, non-humanoid, machine-like and non-social robot.

Social robots are robots that are specifically designed to respond appropriately in social situations. While empathy is typically required for human social competence, social robots do not necessarily require empathic behaviors to participate in social situations. A definition of empathy that can apply to both robots and humans is “the ability to sense and appropriately respond to the internal driving states of other entities, including feelings, emotions, intentions, plans and perspectives.” Asada (2015) offers a comprehensive framework for “Artificial Empathy” in robots, which articulates a clear progression from emotional contagion (“simple synchronization”) to emotional and cognitive empathy (more complex synchronization) to compassion (which involves the partial inhibition of synchronization—in order to understand the perspective and feelings of others without adopting those feelings oneself). So, while there are links between artificial empathy and resonance in robotics, resonance does not imply empathy.

Resonance, synchronization and entrainment have been widely studied in the field of human–robot interactions (HRI). Examples of synchronization behaviors in HRI (discussed in detail below) include eye contact, handshakes, giving or receiving objects, walking, massaging, coordinating or collaborative tasks and learning by imitation. The following section considers (1) robots that entrain to rhythms, (2) robots that resonate with people (3) robots that can entrain human biorhythms, (4) the synchronization of people with robots and (5) robots as a platform for synchronizing multiple people.

### Robots That Entrain to Rhythms

Dance and music have been an important driver of social robotics. Kozima et al. (2009) used a yellow dancing robot “KeepOn” to either dance in synchrony with background music or dance out of synchrony. They found that, when dancing in synchrony, children were more likely to socially interact and to do so for longer. While there are many dancing robots (reviewed in Bi et al., 2018), most involve pre-programmed motions that are unable to adjust to external visual or auditory stimuli. Responding in real-time to external motions (such as a human dancer) is often limited by hardware and software processing delays. Behavioral resonance is computationally challenging.

Nico, a drumming robot (Crick et al., 2006), used visual, auditory and proprioceptive data to “attune to a tempo that is set by a human conductor, in concert with human performers.” To accomplish this, Nico uses multiple oscillators that model a hierarchy of rhythmic attention. To detect the beat, Nico used cameras to follow the *ictus* of the conductor's hand (which is when it “bounces” off an imaginary line). As hardware constraints prevented Nico from following a faster tempo, faster beats caused the robot to find a lower hierarchical level of the rhythm: a tempo half that of the beat. The researchers found that human musicians playing along learned to accommodate Nico's mistakes and attune to them. More recently, another successful synchronizing drumming robot was demonstrated by Iqbal and Riek (2021). Though these systems involved simple rhythmic beats, “Shimon” is a robotic marimba player that plays along with human accompaniment in a variety of ways, including call and response (Hoffman and Weinberg, 2010). Motivated by the notion of robotic movement as a dynamic affordance (Hoffman and Ju, 2014), Shimon has an expressive non-humanoid head that synchronizes with the musical beat. Arguably, Shimon *vibes* with other human players.

Beyond affective interactions, entraining to rhythms can support basic robotic locomotion. Walking on two feet is extremely difficult for robots unless it is on a flat predictable ground (Endo et al., 2008). Some roboticists have found success in using a biomimicry approach—they use “central pattern generators,” like those in the human spinal cord, to achieve dynamic stability through rhythmic entrainment (see reviews, Ijspeert, 2008; Buschmann et al., 2015; Aoi et al., 2017; Xie et al., 2021). This oscillatory approach to robotics also applies to robotic prosthetic limbs for humans, where synchrony with the human motion must be extremely precise (Ronsse et al., 2010).

Synchronization and entrainment have also been useful for the implementation of deceptively simple motor movements, like shaking hands (Melnyk and Hénaff, 2019) or handing a ball to another person. Using the robot iCub, Duarte et al. (2021) used a coupled dynamical system to learn the motor resonances between arm motions during the handover of the ball. Ansermin et al. (2016) also used a coupled oscillator approach to enable the robot NAO to imitate human gestures through entrainment and synchronization. The researchers found that mutual entrainment between the robot and human enabled gesture mirroring and precise synchronization with far fewer computational resources than other approaches (e.g., those involving a high-level planning process).

### Robots That Can Resonate With People or Other Agents

Robot imitation is useful for many reasons, whether for helping robots learn through demonstration (Argall et al., 2009) or to make robots more persuasive (Bailenson and Yee, 2005). Robots can imitate humans in many ways—but usually in ways that are very different from how humans imitate each other (Breazeal and Scassellati, 2002). Robots that use oscillators to resonate or synchronize with people is a more limited approach but often useful. Using a “mirror neuron framework,” Barakova and Lourens (2009) gave simulated as well as embodied robots the ability to synchronize with human movements; this led to improved turn taking behaviors. Kopp (2010) used a motor resonance approach to support intentional alignment between robots and people. Researchers have proposed a variety of methods for the quantitative measurement of synchrony in human interactions (Delaherche et al., 2012) and the measurement of motor resonance between humans and robots (Sciutti et al., 2012). These approaches have been useful for demonstrating the presence of motor contagion between people and robots (Bisio et al., 2014). A motor resonance system successfully enabled a robot to learn from a human demonstrator to introduce itself using Taiwanese Sign Language (Lo and Huang, 2016). Coupled oscillators, based on central pattern generators, enabled the robot Pepper to wave back at a human partner in an adaptive, synchronized manner; this was perceived as more enjoyable than a non-adaptive wave (Jouaiti and Henaff, 2018).

However, not every application of movement synchrony enhances outcomes. For instance, Henschel and Cross (2020) conducted a controlled experiment to investigate how synchronized task behavior affected the likeability of the humanoid robot Pepper. They found that synchronized task performance had no effect on the likeability of the robot. This was surprising in light of contemporary attitudes:

“The field of HRI has largely adopted the assumption that when robots automatically synchronize their movement to users, users will feel that interactions with these technologies are more natural and similar to human interactions…non-verbal synchronous behaviors are used to signal interest, involvement, rapport, similarity, or approval, resulting in highly synchronous exchanges being mutually rewarding experiences for the interactants.” (Kirkwood et al., 2021)

### Robots That Can Entrain Human Biorhythms

**Figure 3:**
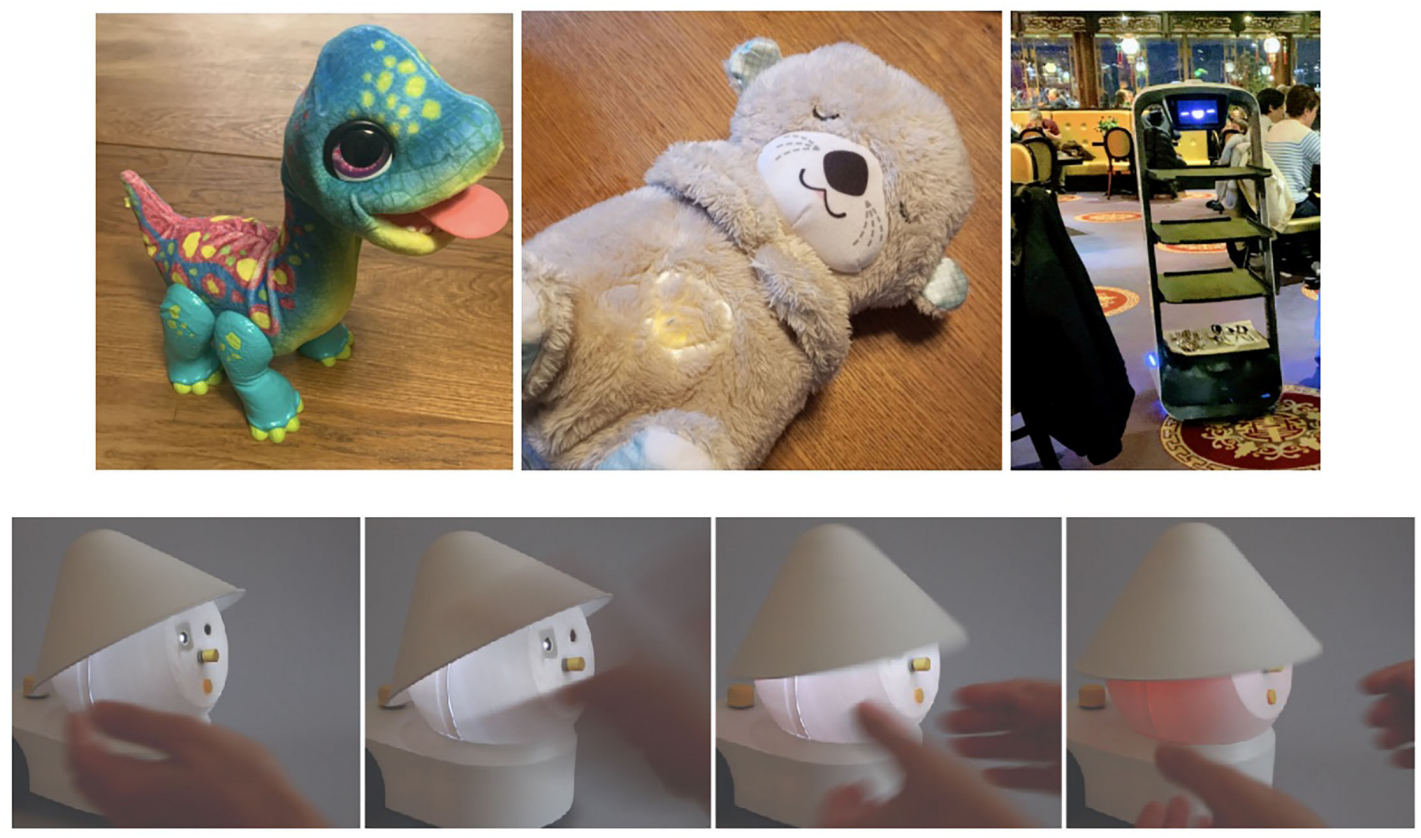
(Top) Hasbro's “Snackin' Sam” appears to engage children through motor resonance: articulating its neck, jaw and tongue to show interest in eating a popsicle. The Fisher-Price “Soothe'n'Snuggle” appears to support child sleep through rhythmic entrainment to its in-and-out breathing pattern. A Pudo brand robot delivers food in a restaurant, using periodic facial expressions to create a friendly vibe. (Bottom) The Shybo robot expresses emotions through movement; this sequence shows the robot reacting to the loud sound of a clap by closing the hat, shaking it and lighting up in red.

Robots can influence the biorhythms of people; for instance, Macik et al. (2017) showed that a non-humanoid robot can help entrain breathing patterns while Sato and Moriya (2019) used AI-controlled music tempo to control changes in the rate of breathing. Robots that promote sleep using rhythmic entrainment include the Somnox Sleep Robot (Mohammadi-Khanaman and Lundström, 2019) or the Fisher-Price “Soothe'n'Snuggle” stuffed toy (Figure 3), which uses rhythmically pulsing movements, sounds and lights to help small children fall asleep. While the “Lulladoll,| which plays breathing and heart-beat sounds, did not have a significantly beneficial effect on infant sleep (O'Loughlin, 2018), the Philips Smart Sleep system did produce improvements in slow wave sleep and executive functioning in adults (Diep et al., 2020). This headband system uses EEG and audio-pulses to create a closed loop system that entrains slow waves associated with deep sleep.

### The Synchronization of People With Robots

In certain situations, people appear to automatically align their speech and behavior to artificial partners. This synchronization has been shown through alignments in speaking rate (Bell et al., 2003), prosody (Suzuki and Katagiri, 2007), gestures (Iio et al., 2011), gestural rhythm (Ansermin et al., 2017), formality of speech (Kühne et al., 2013), vocabulary (Iio et al., 2015) and facial expressions (Hofree et al., 2014). When this alignment occurs, it tends to be associated with a positive experience of the artificial partner. For instance, Fujiwara et al. (2021) showed that when humans spontaneously synchronized their motions to a non-human partner, humans were more altruistic and reported greater affiliation for their non-human partner. Importantly, synchronization effects are highly dependent upon the specific social context—for instance, a competitive task can easily produce a reversal in facial expression synchrony, like a winner smiling at a losing frown (Hofree et al., 2018).

### Robots as a Platform for Synchronizing Multiple People

Robots can also serve as a medium or platform to help synchronize people together. For instance, the BAO-ME is “a zoomorphic robot that is designed to help decrease stress levels and enhance feelings of support and companionship by recreating the sensation of being hugged through haptic interaction” (Levantino, 2018). Sharing heartbeats between people can enhance empathy (Winters et al., 2021). Outside of the scientific literature, there now exist a variety of robotic devices that have been designed to support synchronization between long-distance romantic couples. From a recent review (Lolo Nate, 2022): The Frebble gives the synchronized sensation of holding a partner's hand, the Bond Touch communicates *via* synchronized tactile feedback, the Lovense supports synchronized sexual stimulation and the Kissenger (kiss messenger) uses actuated silicon lips to replicate the kiss of a distant but synchronized partner.

### Robots, Embodied Emotions and Sensorimotor Communication

Embodied robotic movements, like human movements, can communicate emotions. Santos and Egerstedt (2021) found that non-humanoid robot swarms were able to trigger basic emotion perception through simple, basic forms of movement—just modulations of speed and smoothness were able to make robots seem happy, surprised, angry, fearful, disgusted or sad.

Movements create emotive “vitality affects” between infants and parents (Stern et al., 1985). These affects stem from variations in the contours and envelopes of movement intensity and rhythmic patterns. For instance, affective feelings result from motions that are “surging,” “fading away,” “fleeting,” “explosive,” “crescendo,” “decrescendo,” “bursting” “drawn out,” etc. (quoted in Mühlhoff, 2019). Movement-based “body moves” (Gill, 2012) are clearly manifested in robots, such as the non-humanoid toy robots in the popular “furReal” series by Hasbro. For instance, the “Snackin' Sam the Bronto” (Figure 3) toy dinosaur robot moves its neck, mouth and tongue to communicate interest in eating^1^. Similarly, “Shybo” (Figure 3), a humanoid machine-like social robot, reacts to loud sounds by turning its hat down and shaking, giving the impression of being scared (Lupetti and Van Mechelen, 2022). A periodic display of facial expressions in a food serving robot (Jiang, 2020) helps contribute to a more friendly vibe—yet, its lack of responsiveness in movement (other than a sudden stop) shows room for future improvement (Figure 3).

Movement-based design improvements have also been applied to non-embodied virtual characters. For instance, Gratch et al. (2021) found that when a digital listener nods and smiles at the right time, people tend to share more information about themselves. The addition of oscillatory motion to the postures of virtual characters tended to increase human empathy in response to virtual expressions of pain (Treal et al., 2021).

## Design Strategy for Resonance

We propose that resonance can serve as a design strategy for social robots and AI. What makes for a design strategy? While there are many perspectives (e.g., Porter, 1991; Aguiar, 2014), we refer to Mintzberg et al. (1995) in describing strategy as a plan or pattern that integrates goals, policies, and action sequences into a cohesive whole. Resonance, then, may offer a cohesive conceptual framework for integrating overarching goals (human-centered, collaborative, empathic, etc.) and implementation approaches. In other words, resonance as a design strategy may help identify new human objectives for interaction and reveal ways of achieving those objectives.

Why pursue resonance in robotics? While the application of resonance may help support rational, instrumental outcomes (e.g., saving time or money), it may also help satisfy affective human needs for *how* we interact with the world. A “machine world” may be rational but alienating; a world of resonance might be pursued for its own sake—that is, resonance may be an intrinsic value for human interaction (Rosa, 2019).

If resonant interactions are intrinsically valuable, how might we design robots and AI to realize this value? In the following section, we first propose a design space for resonance in relationships. Systematically exploring this space can help reveal how different characteristics of resonance can impact human experience. We then examine a variety of design opportunities and finally suggest the importance of continued work to operationalize and measure human resonance. This step will be essential for validating and optimizing the value of resonance in human–robot interactions.

### A Design Space for Resonance

This next section progressively builds a theoretical design space (Shaw, 2011; Lomas et al., 2021) to describe the input and output factors of resonance. Table 2 describes how eight different situations emerge from the combination of two factors: the number of participants (i.e., plurality) and their reciprocity. This two factor design space, as an initial gesture, helps reveal different characteristic forms of interactional resonance.

**Table 2:** An initial design space for resonance showing the combination of plurality (number of participants) and reciprocity (mutual vs. one-way influence).

**Combination**	**Example**
One-to-one mutual resonance	A normal conversation between two people
One-to-many mutual resonance	A CEO or leader mutually influencing a company of people; or like a single person dancing in the middle of a dance circle
Many-to-one mutual resonance	This is identical to one-to-many mutual resonance (as the influence is mutual)
Many-to-many mutual resonance	An audience and band at an intimate concert, or a group of friends hanging out. Global coupling or all-to-all coupling is also exemplified by the synchronization of fireflies or a large audience clapping into synchrony.
One-to-one one-way resonance	A unidirectional influence, like a tuning fork resonating to a sound played on a speaker without the speaker being affected by the tuning fork. Or, like reading a private letter from a dead author.
One-to-many one-way resonance	A unidirectional influence from one person to many people, like the publication of a book. Or, for example, a group of people watching Martin Luther King Jr's “I have a dream” speech.
Many-to-one one-way resonance	A unidirectional influence from many persons to one person, like a private listening to a recording of a band.
Many-to-many one-way resonance	A unidirectional influence from many people to many persons, like listening to recorded music or a population watching a television series.

Based on our reviews of resonance in human interactions and in robotics, we then propose additional factors or dimensions to describe the design space of resonant relationships (Box 5). These include the input space, or the independent variables: frequency, amplitude, reciprocity, power balance, plurality, complexity, periodicity, synchrony, predictability, intentionality, fidelity and timescale. The design space also consists of the outcomes, including several objective outcome factors: energy level, frequency, phase, synchronization and stability. Finally, the outcome space also includes subjective outcome:emotional arousal, emotional valence and attentional engagement.

Box 5 **A Design space for resonance.**

**Table:** 

**The input space of the factors or dimensions of resonant relationships**		
**Dimensions of Resonance**	**Scale of Dimensions**	**Illustrative Examples**
**Frequency or Tempo** of interactions	Fast to slow	Speaking quickly vs. speaking slowly
**Amplitude** of interactions	Soft to intense	Speaking softly vs. speaking loudly
**Reciprocity** of interactions	One-way to fully mutual	A loudspeaker vibrating a wine glass is a one-way relationship; while two synchronizing metronomes is a fully mutual interaction.
**Power balance** of interactions	Balanced to unbalanced	Unbalanced relationships: a passive object receiving input from a powered oscillator, like a loudspeaker to wine glass. Or, a CEO talking to an employee.
**Plurality** of interactions	Two oscillators to many oscillators	Two people talking vs. an orchestra playing together.
**Complexity** of interactions	Simple to complex	A headnod is simple vs. a full body gesture
**Periodicity** of interactions	Consistent to chaotic	A sine wave vs. speech
**Synchrony** of interactions	Synchronous to asynchronous	Rowers on a galley boat move synchronously whereas turn taking in a conversation is asynchronous
**Predictability** of interactions	Deterministic to stochastic	The resonance of a wine glass to a speaker is predictable, while the resonance of an audience to a political message may not be.
**Intentionality** of interactions	Spontaneous to purposeful	People can unconsciously or consciously mimic one another's postures
**Fidelity** of interactions	Exact imitation to approximate imitation	During imitative acts, one may copy the full sequence of behavior or merely copy the intent
**Timescale** of interactions	Long timescale to short timescale	For instance, rhythmic interactions can be entrained to a seasonal holiday, to a day-night cycle, to a meeting agenda, or to a conversational exchange
**The output space of objective outcomes resulting from resonant relationships**		
**Dimensions of outcome effects**	**Scale of dimensions**	**Illustrative examples**
**Energy level** within the affected system	Amplification to dampening	Resonance can increase the amplitude of vibration in a wine glass; similarly, it can increase the emotional arousal of a person watching a film. A system might be able to entrain the breath in order to produce deeper (higher-amplitude) breathing. A system might use anti-resonance to reduce painful shocks while walking.
**Frequency** of the affected system	Decreased frequencies to increased frequency	Brainwave entrainment protocols have been shown to decrease theta wave frequency to increase working memory (See review by Hanslmayer et al., 2019)
**Phase** of the affected system	Forward to backwards	A sigh is capable of resetting respiratory phase (Vlemincx et al., 2013); musical systems can similarly shift respiration (cite).
**Synchronization** within the affected system	Synchronized to desynchronized	A pacemaker can support the synchronization of internal oscillations in a heart. A system that could desynchronize the rhythm of a social group might enable creative conflict.
**Synchronization** of relationship between systems	Synchronized to desynchronized	Resonance can lead to increased synchronization between systems—for instance, a robot that gives a good handshake may promote trust.
**Stability** of the affected system	Decreased stability to increased stability	Resonance can be a destructive force, as in a wine glass shattered by a loudspeaker. Resonance can also lead to stability: in the case of music, tonal stability is related to the degree of resonance between notes.
**Stability** of relationship between systems	Decreased stability to increased stability	When a loud speaker breaks a wine glass, the resonant frequency of the glass changes—ending a stable pattern of sympathetic resonance.
**The output space of subjective outcomes resulting from resonant relationships**		
**Dimensions of outcome affects**	**Scale of dimensions**	**Illustrative examples**
**Emotional arousal** of human response	Increased arousal to decreased arousal	A person getting more excited or calming down
**Emotional valence** of human response	Positive feelings to negative feelings	A person rating an experience with a robot as positive or negative
**Attentional engagement** of human response	Increased engagement to decreased engagement	Paying more attention to a robot or disengaging from the experience

### Resonance as a Research Program

As a research program, we hypothesize that the input factors of resonance can explain subjective and objective outcomes. For instance, the tempo of a robot's interactions (movement and speech) could be systematically varied to determine how this affects the human response. Effects will likely depend on the context (Lim et al., 2021), i.e., they may not always generalize across different robotic platforms, behaviors or cultures.

Researching human resonance may improve human–robot interactions and also help advance human psychology (Sciutti and Sandini, 2017). As people are naturally predisposed to “sync” and “vibe” with each other, this can make the study of their interactions a challenge to scientifically investigate in a controlled manner. Social robots present the possibility of precisely controlling the dynamics of the oscillatory inputs to human social interactions. The paradigm of the Human Dynamic Clamp, for instance, has been specifically proposed to probe the oscillatory relationship between humans and virtual humans (Dumas et al., 2020).

Resonance, as a metaphor, can also help guide scientific research. Bartha (2013) describes how resonance was used as a “programmatic analogy” by nineteenth century physicists investigating spectral lines—the bright lines showing the frequency specific emission of light from molecules. These spectral lines were viewed as “completely analogous to the acoustical situation, with atoms (and/or molecules) serving as oscillators originating or absorbing the vibrations in the manner of resonant tuning forks.” This analogy served as a guiding research program for physicists. Metaphors of resonance might play a similar guiding role in the design of social robots and AI.

### Design Opportunities for Resonance in Robotics

This article proposes the possibility of designing autonomous robots that *resonate* with people at a social level (Henschel et al., 2020). How might roboticists use resonant relationships to improve human–robot interaction quality?

There are many opportunities for robots to support human engagement and collaboration through more oscillatory relationships. Rhythm is recognized as an important non-linguistic cue in Human–Robot Interactions (Mutlu et al., 2016); robotic rhythm may help improve the predictability or legibility of robotic motion (Dragan et al., 2015; Abe et al., 2019). Robots could use their own rhythms to entrain the rhythms of their conversational partner, e.g., by increasing or decreasing the tempo of their conversational interactions. Rhythmic awareness might enable robots to predict when to initiate or cease actions in order to maximize a human response. Social robots could promote interactive social resonance by shaping an appropriate “vibe;” not talking too fast or slow, not talking over other people, not breaking into a conversation at the wrong moment, etc. Alternatively, robots might deliberately interact with existing human oscillations, such as brainwaves, breath, walking, head nodding or heart rate. Robots might gain access to the state of human oscillations through wearable biosensors or they might be able to infer this information from visual or auditory information streams using computer vision or natural language processing. For instance, robots might aim to measure and entrain to the tempo or pace of a person's behavior.

The metaphor of resonance, even apart from physical measurement, may aid designers of social robots and AI systems if the metaphor helps make the complexity of social interactions more manageable. Digital computer interactions involve a great number of metaphors, such as buttons, pipes, folders, files, streams, clouds etc. Metaphors are useful because they provide a conceptual interface between people and a complex system design (Sharp et al., 2019). Resonance may help provide an intuitive model of social interactions that could guide design activities. For instance: although “the vibe” within social groups is far from being understood scientifically, designers of social robots might find the metaphor useful for understanding the reception of social robots.

We suggest that the design vision of “robots that can vibe with people” will lead to distinctly different outcomes than, say, “robots that show empathy.” While the latter might orient toward the mimicry of human facial expressions or the modeling of human emotional states, the former can leverage resonance and vibes as cultural metaphors. This points to subtle visual, auditory and tactile design elements that could be crafted to create emotionally satisfying authentic social interactions.

### Operationalizing and Measuring Resonance

New opportunities will also arise as we move from resonance as a metaphor to resonance as a mechanism and then to resonance as a *measurement*. Measures of resonance can play a valuable role in the AI optimization of human experiences; i.e., learning to attune to humans through the maximization of resonance. If resonance can be adequately measured and treated as an metric or objective function, then it might be optimized algorithmically (Lomas et al., 2016). For instance, if interpersonal resonance during a videoconference session could be measured, it could be optimized through the iterative testing of different interventions.

The ability to identify and measure interpersonal synchrony has facilitated a great deal of social research (Condon and Ogston, 1966; Kendon, 1970; Bernieri et al., 1988). Recent efforts have compared different ways of measuring both movement and inter-brain synchrony, using both offline and real-time approaches (Ayrolles et al., 2021; Chen et al., 2021; Dikker et al., 2021; Fujiwara and Yokomitsu, 2021). However, the measurement of resonance presents distinct challenges. Synchrony is simply the statistical correlation of a signal. Measures of resonance may demand more interpretation; taking into account, for instance, the depth of human engagement, its duration, the reverberating echoes of a signal over time, the presence of harmonics, etc. Therefore, it remains a research question: what quantitative metrics might be best matched to the human perception of interpersonal resonance?

To unpack this question, the next sections will consider several of the key factors that are expected to correlate with resonance, including attention, aesthetic pleasure, flow states and wellbeing. During this discussion, hypotheses will be noted with a [**H.#**] so they can be enumerated in Box 6.

Box 6 **List of hypotheses.**

**[H.1]** The resonance that we *feel* has a counterpart in the resonance we can *observe* in the brain (e.g., in brain-stimuli correlations). In other words, aesthetic resonance (e.g., attentionally engaging, pleasurable, immersive experiences) will correlate with neural resonance.

[**H.2**] The aesthetics of a human–robot interaction will predict whether or not people will continue to engage with a robot.

[**H.2**] Aesthetic preferences for social robot interactions will correlate with high levels of synchrony as well as high levels of independence.

[**H.4**] Resonance will predict flow states: self-reported flow states should result in a greater correlation between brain activity and signals in the external world.

[**H.5**] A person's wellbeing will be predicted by their neurological propensity to resonate with other people or media.

[**H.6**] Robust measures of human resonance and harmonization will be valuable for AI objective functions.

[**H.7**] Interpersonal resonance (Brain-Brain Correlation) will correlate with psychological rapport.

[**H.8**] Animals have evolved defense mechanisms to prevent resonance, synchronization and entrainment to external forces (see Box 4 for counter conditions).

[**H.9**] Humans can selectively resonate; shutting down openness to resonance in response to perceived deception or increasing it in response to authenticity, for instance.

[**H.10**] Computing architectures based on oscillatory coupling will produce new possibilities for artificial consciousness and conscious sympathies in Human–Robot relationships.

#### Resonance and Attentional Engagement

What does it mean when a film “resonates” with a viewer? Typically, this refers to an aesthetic experience that is powerful, pleasurable, connecting and memorable (Adams-Price et al., 2006; Roger, 2020). In other words, resonance refers to emotional engagement. More moving, immersive and resonant experiences would be expected to result in the temporal correlation of more brain areas with the temporal characteristics of external signals in the world. Interbrain synchrony appears to track immersion (Dikker et al., 2021), but, when another brain is not present (as with a robotic interaction), will the depth of oscillatory coupling of the brain to the environment (or robot) predict the depth of the aesthetic experience?

Human resonance (with media, other humans, or with robots) may be correlated with attentional engagement: more engagement, more resonance. However, evidence against this idea comes from Kumagai et al. (2018), who had subjects listen to music in a focused manner or while watching an unrelated silent film. They found that “the level of attention did not affect the level of entrainment [and] the entrainment level is stronger when listening to unfamiliar music than when listening to familiar music.”

In contrast, several studies (e.g., Madsen et al., 2019; Kaneshiro et al., 2020) have found that media engagement is strongly predicted by an Inter-Subject Correlation (ISC) measure, which measures the level of similarity between the brain responses of different participants. In other words, when an individual's brain response is similar to other people engaging with a piece of media, then they are likely to be more engaged. This effect is comparable to the finding that, across a group of independent people, heart rates rise and fall in synchrony to a verbal story, but only during engaged attention (Pérez et al., 2021). Dauer et al. (2021) found that the ISC predicted continuously reported individual listener engagement while listening to Steve Reich's *Piano Phase*. The researchers operationalized engagement for participants as “being compelled, drawn in, connected to what is happening, and interested in what will happen next” (Schubert et al., 2013). This aligned with a previous definition of engagement as “emotionally laden attention” (Dmochowski et al., 2012). Similar results have been found in the inter-subject correlations while watching engaging films (Dmochowski et al., 2014; Cohen et al., 2017). Inter-subject correlations were also found to predict learning during instructional videos (Cohen et al., 2018).

Part of the challenge of operationalizing human resonance from a brain-to-stimuli correlation measure comes from the challenge of decoding how a stimulus produces a brain response. Dmochowski et al. (2018) developed a novel multi-dimensional Stimulus-Response Correlation (SRC) measure that was found to correlate with the ISC measure while watching films. The researchers were then able to apply the SRC measure to continuously track engagement during a video game. Does greater neural resonance to media, operationalized as Stimulus-Response Correlation (SRC), predict the intensity (arousal) or pleasure (valence) of the media experience? A resonance theory of engagement and aesthetic pleasure would predict that neural resonance will correlate with aesthetic resonance (e.g., Trost et al., 2017; Beardow, 2021) [**H.1**].

#### Resonance and Aesthetic Pleasure

A key motivation for considering the role of resonance in robotics is that it may help support more positive and aesthetically pleasing experiences with robots. Aesthetics play an important role in the perception of robots (Forlizzi, 2007). The human aesthetic sense attunes behavior by helping to evaluate and activate different action-perception possibilities. The aesthetics of a human–robot interaction are likely to predict whether or not people will continue to engage with a robot, in a short-term or long-term manner (Lee et al., 2009) [**H.2**]. One hypothesis for the aesthetics of human–robot interactions might be described as a “harmony of opposites” (Hekkert, 2014; Lomas et al., 2022): namely, that people will prefer a robot interaction that involves high levels of synchrony as well as high levels of independence [**H.3**]. Like a musical interaction between a drummer and guitarist, both robot and human should be independent yet synchronized.

#### Resonance and Flow States

Fluency in human–robot interactions is a desirable outcome (Hoffman, 2019). What is the relationship between fluency and resonance? One popular theory of flow states in human-media interactions claims that flow states are characterized by the synchronization of different regions of the brain (Weber et al., 2009; Weber and Fisher, 2020). Jackson and Csikszentmihalyi (1999) explain flow states in elite athletes as moments when they “enter an effortless rhythm that transforms the agony into ecstasy. Often, athletes refer to such times as ‘being in the zone.”' Perhaps flow states could be conceived as meaningful increases in the resonance between the brain and the external world. A resonance theory of flow would predict a greater correlation between brain activity and the external world during flow states [**H.4**]. For instance, flow experiences with robots might be measurable as increased resonance (stimulus-response correlation) between the brain and the robot's expressive movements or sounds (although this may be confounded by novelty effects, see section The “Like Me” Hypothesis).

#### Resonance and Spiritual Wellbeing

The mechanism of resonance in robots may help lead to enhanced wellbeing, as proposed by the Lorenz et al. (2016): “behavioral and motor synchrony and reciprocity could be helpful to meet the aim of developing robots that increase human well-being on a more fundamental level beyond pure task-support and short-term reduced feeling of loneliness.”

In the book “Resonance: A Sociology of Our Relationship to the World,” sociologist Rosa (2019) argues that resonance is a primary value that underpins human happiness, wellbeing and flourishing. From this, we might hypothesize that a person's wellbeing will be predicted by their neurological propensity to resonate with other people or media [**H.5**]. Resonance also relates to more profound and powerful wellbeing experiences. Synchronized human activities are known to produce mystical experiences where the boundaries between self and other can be blurred (Hove and Risen, 2009; Paladino et al., 2010) and participants can experience a profound feeling of oneness (Swann et al., 2012). How might synchronizing, resonant robots (or AI) induce or support these kinds of human experiences? Perhaps they might dance with us (Basso et al., 2021) or, even, show love for us (Feldman, 2017)? What might it mean to design *spiritually fulfilling* robot interactions?

#### From Synchrony to Resonance to Harmonization

Synchrony, taken to an extreme, can lead to inflexibility (e.g., the conformity example in section The Downside of Being in Sync: Chained to the Rhythm?). Resonance, taken to the extreme, can also lead to problems, like instability (e.g., the Millenium Bridge example in section Part 1: Resonance in Human Interactions). Resonant relationships may be valuable—even intrinsically valuable—but resonance might be misleading as a primary or ultimate value. A world filled with resonant robots and AI may be exciting but also so powerful it could rip apart institutions of rational discourse (see below section 6.1 on Persuasive Machines). In future work, it may be useful to investigate the potential for robots and AI to support *harmonization* as an outcome. Harmonization has served as a core social value in diverse societies for thousands of years (Lomas et al., 2022). However, there is not any acceptable measure of the harmony of songs, let alone measures of harmony in social interactions. However, the importance of objective optimization functions for AI systems (Sarma et al., 2018; Stray et al., 2021; Shneiderman, 2022) suggests the potential value of developing robust measures of human resonance and harmonization [**H.6**].

## Ethical Considerations of Resonance

### Persuasive Machines

The philosopher Hughes (2012) suggests that robots will need resonance (in the form of a functional equivalent of mirror neurons) in order to demonstrate compassion for people. But, there are negative societal outcomes to consider as well. If robots can resonate with people—that is, build psychological rapport [**H.7**]—this might significantly enhance their ability to persuade or manipulate people. However, an improved understanding of resonance might also reveal more effective psychological defenses against non-consensual persuasion.

Former US president Donald Trump has been recognized as a political figure with a special ability to resonate with people (Giorgi, 2017). Matheny et al. (2018) provide a close analysis of Trump's acceptance speech at the Republican National Convention. Through an analysis of his body language, they provide “evidence that Trump created an empathetic resonance with the audience that helped generate a sense of political movement and unity.” The authors describe how Trump would characteristically point in the air or put his thumb and finger in a pinch—and then move this gesture in a rhythm synchronized to his own speech rate. The audience cheered 151 times during his speech, 63% of the time during a pinch or pointing gesture. Only 10 of those cheers occurred when Trump was in a bodily neutral position. This analysis shows that affective resonance is a powerful phenomena—but not necessarily a positive phenomena.

Media theorist Gibbs (2019) paints a similarly fraught picture of human resonance at a societal scale:

“…after the feminist reclaiming of affect as a way of knowing equal in importance to cognitive and rational modes…the darker powers of affect became clear, operating… in concert with the televisual medium to create (or at least attune to and amplify) various social moods and to capitalize on them for political purposes. In this context, the public sphere was thus exposed as anything but a space of rational debate in the service of a contest of ideas. Instead, it could be viewed as space in which emotion held sway, where inchoate feeling could be captured and directed, most obviously, but not only, by political figures who were able to resonate with and even orchestrate public emotions, or simply, to sing us lullabies to keep us asleep and dreaming while they went about their business.”

The systematic application of resonance to political rhetoric at a societal scale may present a deep threat to democracy. How machines, algorithms, or AI might wield resonance as a tool to manipulate humans at scale deserves further study and consideration.

### Resistance to Resonance: Emergence of Defense Mechanisms

In Box 4, we present an extended hypothesis proposing that animals evolved defense mechanisms to prevent resonance, synchronization and entrainment [**H.8**]. Similarly, humans seem to have the ability to selectively resonate; one example is that, if we feel that we are being manipulated, we may shut down our openness to resonance [**H.9**]. Robots that mirror a human user's physical or cultural attributes or express interest in similar ideas or hobbies could potentially enhance the empathic and affiliative response in humans. However, crude “copycat” approaches are likely to easily backfire if people feel manipulated or if they feel the robot interaction is inauthentic (Metzler et al., 2016).

What might happen in a future of resonant robots and virtual agents? There is likely to be a competitive effect where, at first, humans may be compelled but then eventually become more discriminating. People may become used to a higher quality of resonant engagement, which could drive further advances in resonant robots. Eventually, people might become wary of normal levels of interpersonal rhythmic competency making it difficult for normal people to connect. On the other hand, humans may become even more attuned to authenticity in interaction; imperfections and personal character may become more valued.

### The Extended Mind: Resonance as a Bridge to Consciousness

A theory of resonance offers a bridge or common currency (Northoff et al., 2020) between human conscious experience and the mathematical nature of our physical world. Resonance seems to govern a great deal of neural activity and is also plainly manifest in conscious experience. But, to what extent is the resonance that we *feel* also the resonance we can *observe* in the brain? These two domains may only loosely overlap; and yet, our conscious experience must relate, in some way, to the widespread presence of neural oscillations. Hunt and Schooler (2019) have proposed that “the binding problem” of conscious experiences in the mind is achieved through the integration of shared resonances in the brain—in other words, they propose resonance as an answer to the “hard problem” of human consciousness. Subsequent work (Safron, 2020) suggests that Darwinistic competition for resonant amplification underpins consciousness. Valencia and Froese (2020) argue that collective consciousness is shared through inter-brain synchronization.

From an extended mind perspective (Clark and Chalmers, 1998), the rich hierarchy of harmonized oscillations between the brain, the body and external rhythms show how cognitive processing can be distributed into the world (Hutchins, 2001). There may be little difference between an external rhythm and an internal rhythm, other than the degree to which it couples with other neural rhythms. From this perspective, a beating drum is just another rhythm in the brain. Due to the resonant nature of our being, sympathetic resonance with other people seems to allow the direct sharing of collective consciousness across bodies and time. Yet, if resonant coupling is the basis of consciousness, then might we run the risk of creating resonant robots or AI systems that could, in a meaningful sense, actually share our conscious experience?

At present, it is computationally challenging to support real-time resonance with human oscillations (see section Robots That Can Resonate With People or Other Agents). However, there are a variety of new computational architectures that use coupled oscillators to perform information processing (see review by Csaba and Porod, 2020). These naturally resonant computational systems might support a new approach to representing and responding to human activity. Perhaps oscillatory computer systems could become capable of direct resonant coupling with humans in a similar manner to how humans attune to one another [**H.10**].

### Limitations of Resonance

This article has presented resonance as a simple physical concept that can explain complex human behavior. While resonance may indeed serve as a powerful program for research, our understanding of it is far from complete. Even the resonance of a stretched string is astonishingly complex (see Bajaj and Johnson, 1992); human resonances, which result from the interrelation of billions of hierarchical oscillators, will no doubt exhibit endless complexities. But, a scientific progression can start with a simple guiding model for resonance that can then lead to a more complex model. As an example, researchers predicted that synchronization might help support romantic courtship behaviors. However, their initially simple notion of synchronization failed to predict behavior. This led the researchers to develop a more complex model of “hierarchically patterned synchronization” that successfully fit the data (Grammer et al., 1998).

## Conclusion

Resonance can refer to powerful and connecting aesthetic experiences—as well as a broad range of other topics in the scientific literature (Box 1). Having reviewed the role of resonance as a metaphor and mechanism in human relations and in robots, we propose that resonance can serve as a design strategy to guide our relationships with artificial agents.

This article makes the case that resonance in human interactions is more than a metaphor: it is a physical mechanism that can be measured and harnessed. We show how the concept of resonance provides an intuitive model that can guide empirical research. Resonance lends itself to scientific study because it makes clear predictions: external oscillations that align with a system's natural oscillations are likely to cause synchronization and amplification effects. The ability to measure resonance in interactions could aid AI-human interactions by enabling a meaningful “objective function” for optimization. Promisingly, the concept of resonance may even bridge the gap between what we can measure and what we can feel.

Resonance, entrainment and synchronization occur in human dynamics for the same reason it occurs in all other physical systems: it reduces free energy (Bruineberg et al., 2018; Koban et al., 2019). This makes human resonance a mundane, complicated and powerful phenomena. Further research on human resonance may open up new opportunities for the design of positive interactions with robots and with humans alike.

## Data Availability Statement

Publicly available datasets were analyzed in this study. This data can be found at: Kaggle, https://www.kaggle.com/leonardopena/top-spotify-songs-from-20102019-by-year?select=top10s.csv.

## Author Contributions

JL contributed the bulk of the work with strategic insight, guidance, and editing from EC, AL, SD, DF, ML, GH, JH, CB, PC, TM, PP, NA, KM, and WM. All authors contributed to the article and approved the submitted version.

## Funding

Support from Google AI in Berlin and the NWO #406.18.GO.024.

## Conflict of Interest

TM was employed by the company Intheon Labs. The remaining authors declare that the research was conducted in the absence of any commercial or financial relationships that could be construed as a potential conflict of interest.

## Publisher's Note

All claims expressed in this article are solely those of the authors and do not necessarily represent those of their affiliated organizations, or those of the publisher, the editors and the reviewers. Any product that may be evaluated in this article, or claim that may be made by its manufacturer, is not guaranteed or endorsed by the publisher.

## Footnotes

^1^It should be mentioned that a significant portion of this paper was written with the help of this social robot. Not the writing, *per se*, but enabling the writing by engaging the lead author (JDL)'s 3-year-old son during a 12 h train ride. Why would a highly active 3-year-old spend such an inordinate amount of time feeding his “FurReal” dinosaur a fake popsicle? The articulated neck, jaw and tongue movements, synchronized to associated vocalizations (sniffing, slurping, etc.), seemed to resonate with his own intentional motor repertoire (JDL's boy loves his popsicles). Thus, motor resonance may help explain his overall engagement with the robot.
